# Association of Circulating Sex Hormones With Inflammation and Disease Severity in Patients With COVID-19

**DOI:** 10.1001/jamanetworkopen.2021.11398

**Published:** 2021-05-25

**Authors:** Sandeep Dhindsa, Nan Zhang, Michael J. McPhaul, Zengru Wu, Amit K. Ghoshal, Emma C. Erlich, Kartik Mani, Gwendalyn J. Randolph, John R. Edwards, Philip A. Mudd, Abhinav Diwan

**Affiliations:** 1 Division of Endocrinology, Diabetes and Metabolism, St Louis University School of Medicine, St Louis, Missouri; 2 Department of Pathology and Immunology, Washington University School of Medicine in St Louis, Missouri; 3 Endocrine Division, Quest Diagnostics Nichols Institute, San Juan Capistrano, California; 4 LC-MS Core Lab, Quest Diagnostics Nichols Institute, Valencia, California; 5 Cardiovascular Division, Washington University School of Medicine in St Louis, Missouri; 6 Center for Cardiovascular Research, Department of Medicine, Washington University School of Medicine in St Louis, Missouri; 7 John Cochran Veterans Hospital, St Louis, Missouri; 8 Center for Pharmacogenomics, Department of Medicine, Washington University School of Medicine in St Louis, Missouri; 9 Department of Emergency Medicine, Washington University School of Medicine in St Louis, Missouri; 10 Department of Cell Biology and Physiology, Washington University School of Medicine in St Louis, Missouri; 11 Department of Obstetrics and Gynecology, Washington University School of Medicine in St Louis, Missouri

## Abstract

**Importance:**

Male sex is a risk factor for developing severe COVID-19 illness. It is not known whether sex hormones contribute to this predisposition.

**Objective:**

To investigate the association of concentrations of serum testosterone, estradiol, and insulinlike growth factor 1 (IGF-1, concentrations of which are regulated by sex hormone signaling) with COVID-19 severity.

**Design, Setting, and Participants:**

This prospective cohort study was conducted using serum samples collected from consecutive patients who presented from March through May 2020 to the Barnes Jewish Hospital in St Louis, Missouri, with COVID-19 (diagnosed using nasopharyngeal swabs).

**Exposures:**

Testosterone, estradiol, and IGF-1 concentrations were measured at the time of presentation (ie, day 0) and at days 3, 7, 14, and 28 after admission (if the patient remained hospitalized).

**Main Outcomes and Measures:**

Baseline hormone concentrations were compared among patients who had severe COVID-19 vs those with milder COVID-19 illness. RNA sequencing was performed on circulating mononuclear cells to understand the mechanistic association of altered circulating hormone concentrations with cellular signaling pathways.

**Results:**

Among 152 patients (90 [59.2%] men; 62 [40.8%] women; mean [SD] age, 63 [16] years), 143 patients (94.1%) were hospitalized. Among 66 men with severe COVID-19, median [interquartile range] testosterone concentrations were lower at day 0 (53 [18 to 114] ng/dL vs 151 [95 to 217] ng/dL; *P* = .01) and day 3 (19 [6 to 68] ng/dL vs 111 [49 to 274] ng/dL; *P* = .006) compared with 24 men with milder disease. Testosterone concentrations were inversely associated with concentrations of interleukin 6 (β = −0.43; 95% CI, −0.52 to −0.17; *P* < .001), C-reactive protein (β = −0.38; 95% CI, −0.78 to −0.16; *P* = .004), interleukin 1 receptor antagonist (β = −0.29; 95% CI, −0.64 to −0.06; *P* = .02), hepatocyte growth factor (β = −0.46; 95% CI, −0.69 to −0.25; *P* < .001), and interferon γ–inducible protein 10 (β = −0.32; 95% CI, −0.62 to −0.10; *P* = .007). Estradiol and IGF-1 concentrations were not associated with COVID-19 severity in men. Testosterone, estradiol, and IGF-1 concentrations were similar in women with and without severe COVID-19. Gene set enrichment analysis revealed upregulated hormone signaling pathways in CD14^+^CD16^−^ (ie, classical) monocytes and CD14^−^CD16^+^ (ie, nonclassical) monocytes in male patients with COVID-19 who needed intensive care unit treatment vs those who did not.

**Conclusions and Relevance:**

In this single-center cohort study of patients with COVID-19, lower testosterone concentrations during hospitalization were associated with increased disease severity and inflammation in men. Hormone signaling pathways in monocytes did not parallel serum hormone concentrations, and further investigation is required to understand their pathophysiologic association with COVID-19.

## Key Points

**Question:**

Are circulating sex hormones associated with disease severity in patients with COVID-19?

**Findings:**

In a cohort study of 152 patients with COVID-19, including 143 patients who were hospitalized, testosterone concentrations at presentation and on day 3 were inversely associated with disease severity and circulating inflammatory cytokine concentrations in men but not in women. Transcriptional profiling of circulating mononuclear cells revealed upregulation of hormone signaling pathways in patients requiring intensive care vs those with milder disease.

**Meaning:**

These findings suggest that low testosterone concentrations may play a mechanistic role in worse outcomes observed in men with COVID-19, underscoring the need for clinical trials to test this hypothesis.

## Teaser

This cohort study investigates the association of sex hormones with disease severity and inflammatory cytokines in patients with COVID-19.

## Introduction

Coronaviral diseases have constituted a major public health issue during the last 2 decades, starting with the severe acute respiratory syndrome coronavirus (SARS-CoV) pandemic in 2002 through 2003,^1^ continuing with the Middle East respiratory syndrome coronavirus (MERS-CoV) epidemic in 2012,^2^ and most recently, the current COVID-19 pandemic. With a unique combination of transmissibility and lethality, COVID-19 has had a dramatic public health impact. Patients hospitalized with COVID-19 are more likely to be men than women.^3^ This sexual dimorphism has led some to presume that the male sex hormone, testosterone, may be a risk factor associated with the severity of COVID-19 and that estrogen may be protective.^4^ However, testosterone concentrations are highly variable among men and affected by biological variables and pathologic stressors.^5,6^

Testosterone concentrations in men decline continuously by 1% to 2% per year starting after age 30 years.^7,8,9^ In addition, obesity, metabolic syndrome, and many chronic illnesses, such as type 2 diabetes, renal insufficiency, and chronic lung disease, are associated with lower serum testosterone concentrations in men.^5,10,11^ Thus, the severity of COVID-19 illness seems to coincide with the nadir of lifetime testosterone, and the comorbidities that predispose individuals to increased COVID-19 severity are also associated with lower testosterone concentrations. Studies among patients in the hospital, including those with COVID-19, have found that testosterone concentrations are lower in men requiring intensive care unit (ICU) admission or use of ventilators than in those with milder illness.^12,13,14,15^ Men with testosterone concentrations less than the reference range have chronically elevated concentrations of inflammatory mediators.^16,17^ We recently found^18^ that the pattern of inflammation in individuals with COVID-19 differs from that seen in individuals with influenza. Patients with COVID-19, compared with those with influenza, have higher concentrations of a few cytokines (ie, interleukin 6 [IL-6] and interleukin 1 receptor antagonist [IL-1ra]), lower concentrations of most cytokines, and profound type I and type II interferon immunosuppression. We therefore conducted a detailed investigation into the association of testosterone with disease severity and inflammation in patients with COVID-19.

Testosterone is converted to estradiol by aromatase and has a stimulatory effect on the growth hormone axis.^19,20^ There is a decline in estradiol and insulinlike growth factor (IGF-1) concentrations in men and women with age, and lower IGF-1 concentrations have been associated with acute respiratory distress syndrome.^21^ In contrast, higher estradiol concentrations during hospitalization are associated with increased mortality in both sexes.^22^ It is not known whether estradiol and IGF-1 concentrations are associated with disease severity in individuals with COVID-19.

In view of the above, we investigated the association of serum testosterone, estradiol, and IGF-1 concentrations with COVID-19 severity and inflammatory markers. Additionally, we interrogated the signaling pathways by RNA sequencing in peripheral monocytes to understand hormone signaling at a cellular level.

## Methods

The cohort study was approved by the Washington University in St Louis Institutional Review Board, and patients provided verbal consent to participate. This study is reported following Strengthening the Reporting of Observational Studies in Epidemiology (STROBE) reporting guideline.

We used serum samples that were prospectively collected from patients who presented in March through May 2020 to the Barnes Jewish Hospital in St Louis, Missouri, with symptoms suggestive of COVID-19 illness and were confirmed to have SARS-CoV-2 infection on nasopharyngeal swabs with clinical polymerase chain reaction assays. Demographic data, including race (which was self-reported), were collected at time of hospital admission and extracted from clinical charts by a university research team that was not directly involved in this study. The data were made available in an anonymized fashion to us. Racial disparities have been noted in outcomes of COVID-19.^23^ Hence, we included race as a covariate in our analysis. Testosterone, estradiol, and IGF-1 were measured at the time of presentation (ie, baseline or day 0) and at days 3, 7, 14, and 28 after admission (if the patient remained hospitalized). Patients who were not hospitalized had only day 0 data available.

### Hormone Assays

Total testosterone, estradiol, and IGF-1 were measured by liquid chromatography–mass spectrometry by methods previously described.^24^^,^^25^^,^^26^ Details are presented in the eAppendix in the Supplement.

### Cytokine Quantification

Plasma obtained from study participants was frozen at −80 °C and subsequently analyzed using a human magnetic cytokine panel providing parallel measurement of 35 cytokines (Thermo Fisher Scientific), as previously described.^18^ Additional details are included in the eAppendix in the Supplement.

### Peripheral Blood Mononuclear Cell Sorting and RNA Sequencing

Cryopreserved peripheral blood mononuclear cells from 12 male and 8 female patients with COVID-19 were thawed and sorted. This was followed by RNA sequencing analyses as detailed in the eAppendix in the Supplement.

### Statistical Analysis

The primary exposure of the study was baseline testosterone concentrations, and the primary outcome was severe COVID-19, defined as any of the following events at any time during hospitalization: hypoxia requiring supplemental oxygen, need for mechanical ventilation, need for ICU treatment, or death due to COVID-19. The comparisons were adjusted for age, body mass index (BMI; calculated as weight in kilograms divided by height in meters squared), race, smoking history, and comorbidities at baseline using the Charlson Comorbidity Index (CCI) (eAppendix in the Supplement).^27^ These models addressed missing data values by maximum likelihood, under the data missing at random assumption. Sensitivity analyses were performed to evaluate this assumption. Based on these analyses, the missing at random assumption was deemed reasonable. Continuous variables are presented as means and SDs or medians and interquartile ranges (IQRs), depending on the distribution of values. Nonnormal data were log-transformed to conduct parametric tests. All tests were performed using SPSS statistical software version 27 (IBM Corp). Group comparisons were performed using *t* tests, Mann-Whitney rank sum tests, and χ^2^ tests, as appropriate. Results of multivariate linear regression analyses are presented with standardized coefficients (β) and *P* values. Multivariate logistic regression analyses are presented as odds ratios (ORs; ie, exponential of β coefficient with 95% CIs and *P* values). Reported *P* values are 2-sided and considered statistically significant at <.05. Cytokine analyses were adjusted for multiple comparisons using the Benjamini-Hochberg approach and a false discovery rate of 5%.

## Results

Among 152 consecutive patients (90 [59.2%] men; 62 [40.8%] women; mean [SD] age, 63 [16] years) with COVID-19, 143 patients (94.1%) were hospitalized. Patients presented to the hospital a median [IQR] 3 [1-7] days after the onset of symptoms; the most common symptoms were shortness of breath (94 patients [61.8%]), fever (88 patients [57.9%]), and nonproductive cough (84 patients [55.2%]). Smaller proportions of patients had myalgia (38 patients [25.0%]), fatigue (26 patients [17.1%]), gastrointestinal symptoms (ie, nausea, vomiting, or diarrhea; 29 patients [19.1%]), or headache (14 patients [9.2%]). During hospitalization, 37 patients (24.3%) died. Men had a lower mean (SD) BMI than women (27.7 [6.8] vs 33.0 [8.8]; *P* < .001), but their age and hospital outcomes were similar (eTable 1 in the Supplement). Men had higher median (IQR) testosterone concentrations than women (79 [38-181] ng/dL vs 12 [1-21] ng/dL [to convert to nanomoles per liter, multiply by 0.0347]; *P* < .001) but similar estradiol and IGF-1 concentrations. We analyzed the association of hormone concentrations with study outcomes within each sex.

### Men

#### Sex Hormones and IGF-1

Testosterone concentrations were available at day 0 in 76 men, and 68 of those men (89.5%) had concentrations lower than the reference range (ie, <250 ng/dL). Testosterone concentrations at day 0 were inversely correlated with CCI score (*r* = −0.32; *P* = .005) but not age (*r* = −0.20; *P* = .09) or BMI (*r* = −0.11; *P* = .33). Testosterone concentrations were positively correlated with IGF-1 concentrations (*r* = 0.32; *P* = .01) but not estradiol concentrations (*r* = −0.20; *P* = .15) at day 0. Median serum testosterone concentrations decreased during hospital stay (eFigure 1 in the Supplement), reaching a nadir at day 3 and returning to baseline by day 28. There were no statistically significant changes in median estradiol or IGF-1 concentrations during hospitalization (eFigure 2 and eFigure 3 in the Supplement). At day 0, the ratio of estradiol to testosterone, which serves as a surrogate marker of aromatase activity,^28^ was positively correlated with age (r = 0.44; *P* < .001) but not BMI (r = 0.20; *P* = .14).

Among 90 men with COVID-19, 84 men were hospitalized and 66 men had severe COVID-19. Men with severe COVID-19 were older (mean [SD] age, 68 [11] years vs 55 [15] years; *P* < .001) and had more comorbidities (median [IQR] CCI score, 3 [2-4] vs 2 [0-3]; *P* = .02) (Table 1). Their BMI was lower, possibly reflecting sarcopenia due to age and comorbidities. Among men with severe COVID-19, 25 men (37.9%) died. Median (IQR) testosterone concentrations in men with severe COVID-19, compared with men with mild COVID-19, were lower by 64.9% at admission (53 [18-114] ng/dL vs 151 [95-217] ng/dL; *P* = .008), 82.9% at day 3 (19 [6-68] ng/dL vs 111 [49-274] ng/dL; *P* = .006), and 84.1% at day 7 (20 [12-93] ng/dL vs 126 [70-221]; *P* = .02) (Table 2). In contrast, while estradiol and IGF-1 concentrations did not differ between the 2 groups, the ratio of estradiol to testosterone was higher in men with severe COVID-19 at days 0, 3, and 7 (Table 2).

**Table 1.** Patient Characteristics and CRP Concentration

Characteristic	Men			Women		
	With severe COVID-19 (n = 66)	Without severe COVID-19 (n = 24)	*P* value	With severe COVID-19 (n = 37)	Without severe COVID-19 (n = 25)	*P* value
Age, mean (SD), y	68 (11)	55 (15)	<.001	68 (14)	51 (19)	<.001
BMI, mean (SD)	26.7 (6.0)	30.0 (8.0)	.04	32.6 (9.3)	34.3 (8.1)	.45
CCI score, median (IQR)	3 (2-4)	2 (0-3)	.02	2 (2-4)	1 (0-2)	<.001
Ever smoked, No. (%)	35 (53.0)	12 (50.0)	.46	18 (48.6)	9 (36.0)	.32
Race, No. (%)						
White	20 (30.3)	5 (20.8)	.72	5 (13.5)	2 (8.0)	.54
African American	44 (66.7)	19 (79.2)		32 (86.5)	22 (88.0)	
Asian	1 (1.5)	0		0	0	
Other^a^	1 (1.5)	0		0	1 (4.0)	
Duration of hospital stay, median (IQR), d	14 (5-23)	5 (3-10)	.002	10 (6-22)	5 (2-7)	<.001
CRP, median (IQR), mg/dL	14.6 (6.0-21.9)	7.2 (3.1-10.8)	.08	10.8 (5.1-18.9)	7.9 (1.4-13.9)	.13

Abbreviations: BMI, body mass index (calculated as weight in kilograms divided by height in meters squared); CCI, Charlson Comorbidity Index; CRP, C-reactive protein; IQR, interquartile range.

SI conversion factor: To convert CRP to mg/L, multiply by 10.

^a^ Other category includes Native Hawaiian and Pacific Islander individuals and American Indian and Alaskan native individuals.

**Table 2.** Serial Hormone Concentrations in Men

Hormone concentration	Concentration, median (IQR)				
	Day 0	Day 3	Day 7	Day 14	Day 28
Testosterone, ng/dL					
With severe COVID-19	53 (18-114)	19 (6-68)^a^^,^^b^	20 (12-93)	53 (10-95)	102 (26-219)
Without severe COVID-19	151 (95-217)^c^^,^^d^	111 (49-274)^c^^,^^e^	180 (71-229)^c^^,^^f^	NA	NA
Estradiol, pg/mL					
With severe COVID-19	15 (10-23)	12 (7-20)	13 (7-19)	17 (9-23)	13 (10-20)
Without severe COVID-19	15 (11-20)	18 (13-20)	12 (11-13)	NA	NA
Estradiol to testosterone ratio, %					
With severe COVID-19	2.3 (1.0-9.0)	3.6 (1.3-2.9)	2.3 (0.9-1.8)	5.0 (1.1-14.8)	0.6 (0.3-4.8)
Without severe COVID-19	1.1 (0.5-1.9)^c^^,^^g^	1.1 (0.5-1.7)^c^^,^^h^	0.7 (0.5-1.7)^c^^,^^i^	NA	NA
IGF-1, ng/mL					
With severe COVID-19	85 (60-116)	79 (54-116)	75 (50-111)	110 (41-124)	73 (58-107)
Without severe COVID-19	99 (66-153)	50 (16-118)	75 (40-111)	NA	NA

Abbreviations: IGF-1, insulinlike growth factor 1; IQR, interquartile range; NA, not applicable (indicated if there were insufficient patients in a category).

SI conversion factors: To convert estradiol to picomoles per liter, multiply by 3.671; IGF-1 to nanomoles per liter, multiply by 0.131; and testosterone to nanomoles per liter, multiply by 0.0347.

^a^ Significant for comparison with day 0.

^b^ *P* = .004.

^c^ Significant compared with men with severe COVID-19, adjusted for group differences in age, body mass index, Charlson Comorbidity Index score, smoking history, and race.

^d^ *P* = .008.

^e^ *P* = .01.

^f^ *P* = .04.

^g^ *P* = .02.

^h^ *P* = .03.

^i^ *P* = .04.

**Figure 1.**
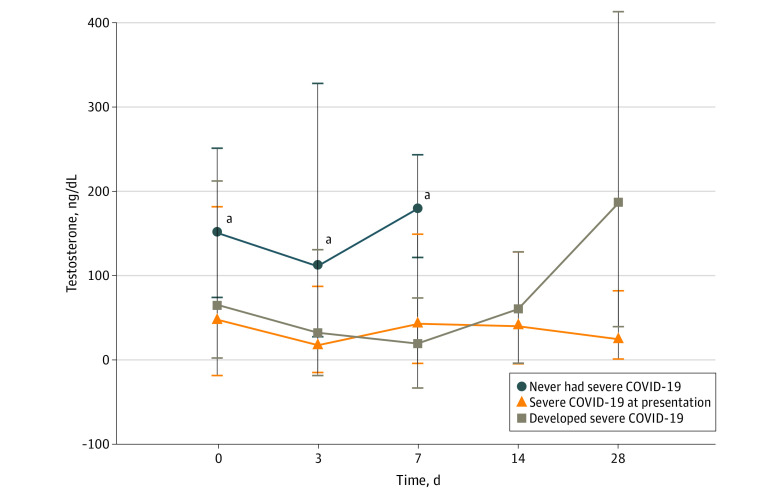
Testosterone Concentration in Men The population included 24 men who never had severe COVID-19, 31 men who had severe COVID-19 at presentation to the hospital, and 35 men who developed severe COVID-19 during their hospital stay. No patient remained hospitalized beyond 7 days in the group that never had severe COVID-19. ^a^Median (interquartile range) testosterone concentrations of men who never had severe COVID-19 were significantly higher than those of men in the other groups at day 0, day 3, and day 7.

Of 66 men with severe COVID-19, 31 men presented with severe disease to the hospital, while 35 men developed severe disease during their hospital stay after a median (IQR) of 2 (1-3) days. Median (IQR) testosterone concentrations upon admission among men who never developed severe COVID-19 (151 [95-217] ng/dL) were higher than those among men who had severe COVID-19 at admission (48 [12-167] ng/dL; *P* = .003) or developed it later during their hospitalization (65 [41-107] ng/dL; *P* = .009). Median (IQR) testosterone concentrations were also higher among men who never developed severe COVID-19, compared with men in the other 2 groups, at day 3 (no severe COVID-19: 111 [49-274] ng/dL; severe COVID-19 at presentation: 18 [7-37] ng/dL; *P* = .002; severe COVID-19 developed later: 32 [7-98] ng/dL; *P* = .007) and day 7 (no severe COVID-19: 180 [71-229] ng/dL; severe COVID-19 at presentation: 43 [15-104] ng/dL; *P* = .04; severe COVID-19 developed later: 19 [12-43] ng/dL; *P* = .03) (Figure 1).

Men who required ICU admission or artificial ventilation or who died had lower testosterone concentrations than men who did not have these outcomes. Median (IQR) testosterone concentration at admission, for example, was 49 (17-109) ng/dL among men who required ICU admission vs 142 (83-221) ng/dL among men who did not (*P* < .001), 38 (10-84) ng/dL among men who required artificial ventilation vs 104 (49-205) ng/dL among men who did not (*P* < .001), and 42 (15-76) ng/dL among men who died vs 108 (49-203) ng/dL among men who survived (*P* = .007) (Table 3). Estradiol or IGF-1 concentrations were not significantly different at baseline or during hospital stay with regards to ICU admission, ventilator use, or mortality status (eTable 2 and eTable 3 in the Supplement). Median (IQR) estradiol to testosterone ratio was higher at admission in men who needed ICU care (3.4% [1.4%-2.5%] vs 0.9% [0.6%-1.9%]; *P* < .001), men who needed artificial ventilation (5.9% [2.0%-185.5%] vs 1.4% [0.7%-2.9%]; *P* = .001), and men who died (3.2% [1.7%-2.7%] vs 1.3% [0.6%-3.2%]; *P* = .009) compared with men who did not have these outcomes. Similarly, median (IQR) estradiol to testosterone ratio at day 3 was higher in men who needed ICU care (10.0% [1.9%-38.4%] vs 1.3% [0.5%-2.9%]; *P* < .001), men who needed artificial ventilation (23.5% [2.9%-131.2%] vs 1.6% [1.1%-4.5%]; *P* < .001], and men who died (12.4% [2.8%-160.0%] vs 1.8% [1.1%-10.0%]; *P* = .01) compared with men who did not have these outcomes.

**Table 3.** Serum Testosterone Concentration in Men by ICU Admission, Ventilator Use, and Mortality^a^

Patient group	Concentration, median (IQR)				
	Day 0	Day 3	Day 7	Day 14	Day 28
With ICU admission (n = 53)	49 (17-109)^b^	17 (5-42)^c^	20 (12-56)^d^	29 (9-90)	27 (24-84)
Without ICU admission (n = 37)	142 (83-221)	104 (49-166)	136 (58-229)	152 (83-221)	230 (215-466)^e^
With ventilator use (n = 24)	38 (10-84)^f^	12 (1-19)^g^	18 (1-35)^h^	15 (7-55)^i^	26 (22-48)^j^
Without ventilator use (n = 66)	104 (49-205)	60 (26-134)	88 (19-195)	93 (81-131)	228 (182-466)^k^
Died (n = 25)	42 (15-76)^l^	15 (1-32)^m^	18 (13-20)	15 (3-45)	NA
Survived (n = 65)	108 (49-203)	49 (14-119)^n^	55 (13-155)	61 (10-110)	135 (26-229)

Abbreviations: ICU, intensive care unit; IQR, interquartile range; NA, not applicable (indicated if there were insufficient patients in a category).

SI conversion factor: To convert testosterone to nanomoles per liter, multiply by 0.0347.

^a^ Comparator group included men with no ICU stay, with no ventilator use, or who survived. Comparison adjusted for group differences in age, body mass index, Charlson Comorbidity Index score, smoking history, and race.

^b^ *P* < .001 for comparator group.

^c^ *P* = .006 for comparator group; *P* =.003 compared with day 0.

^d^ *P* = .04 for comparator group.

^e^ *P* = .04 compared with day 0.

^f^ *P* < .001 for comparator group.

^g^ *P* < .001 for comparator group; *P* = .001 compared with day 0.

^h^ *P* = .001 for comparator group.

^i^ *P* = .01 for comparator group.

^j^ *P* = .004 for comparator group.

^k^ *P* = .007 compared with day 0.

^l^ *P* = .007 for comparator group.

^m^ *P* = .002 for comparator group; *P* = 0.03 compared with day 0.

^n^ *P* = .01 compared with day 0.

Multivariate logistic regression analyses incorporating age, BMI, CCI score, smoking history, and race revealed that testosterone concentrations at day 0 were inversely associated with odds of severe COVID-19 (OR, 0.11; 95% CI, 0.02-0.59; *P* = .02), ICU admission (OR, 0.15; 95% CI, 0.04-0.57; *P* = .007), and ventilator use (OR, 0.29; 95% CI, 0.11-0.81; *P* = .01). Odds were also decreased for mortality, although this difference was not statistically significant (OR, 0.41; 95% CI, 0.16-1.03; *P* = .05). The regression curves of testosterone’s association with outcomes were largely linear, without a clear inflection point (eFigure 4 and eFigure 5 in the Supplement). Age was positively associated with odds of severe COVID-19 (OR, 1.08; 95% CI, 1.02-1.15; *P* = .003), ICU admission (OR, 1.07; 95% CI, 1.01-1.13; *P* = .01), and mortality (OR, 1.10; 95% CI, 1.03-1.18; *P* = .005) but not ventilator use (OR, 1.07; 95% CI, 0.99-1.15; *P* = .08). In these regression models, BMI, CCI score, race, and smoking were not associated with disease outcomes. Testosterone concentrations at day 3 were inversely associated with odds of severe COVID-19 (OR, 0.09; 95% CI, 0.01-0.85; *P* = .04), ICU admission (OR, 0.14; 95% CI, 0.03-0.71; *P* = .018), ventilator use (OR, 0.13; 95% CI, 0.04-0.49; *P* = .003), and mortality (OR, 0.36; 95% CI, 0.15-0.87; *P* = .02).

#### Association of Sex Hormones and IGF-1 With Inflammatory Cytokines

Serum concentrations of 35 inflammatory mediators and C-reactive protein (CRP) were measured on day 0 in 88 patients. The cytokine concentrations did not differ significantly between men and women. After adjustment for age, BMI, CCI score, and multiple testing, men with severe COVID-19 had higher median (IQR) concentrations of serum IL-6 (61 [29-302] pg/mL vs 26 [9-57] pg/mL; *P* = .003) and hepatocyte growth factor (HGF; 994 [383-2308] pg/mL vs 330 [190-467] pg/mL; *P* = .002) compared with men without severe COVID-19. In addition, men who required ventilation had higher median (IQR) concentration of IL-1ra (270 [136-1101] pg/mL vs 95 [54-201] pg/mL; *P* < .001), IL-10 (43 [20-130] pg/mL vs 20 [8-50] pg/mL; *P* = .001), monocyte chemoattractant protein 1 (MCP-1; 1071 [461-1686] pg/mL vs 364 [215-751] pg/mL; *P* = .009), and granulocyte colony-stimulating factor (37 [14-298] pg/mL vs 15 [8-32] pg/mL; *P* < .001). Cytokines were not significantly different in men who survived vs those who died. Median (IQR) concentrations of CRP were higher in men who required ventilation (21.6 [15.1-26.6] mg/dL vs 7.6 [3.7-16.7] mg/dL [to convert to milligrams per liter, multiply by 10]; *P* = .006) or ICU care (17.3 [7.6-24.1] mg/dL vs 6.2 [2.1-10.1] mg/dL; *P* = .004).

On multivariate linear regression analyses using age, BMI, and CCI score, day 0 testosterone concentrations were inversely associated with concentrations of IL-6 (β = −0.43; 95% CI, −0.52 to −0.17; *P* < .001), CRP (β = −0.38; 95% CI, −0.78, to −0.16; *P* = .004), IL-1ra (β = −0.29; 95% CI, −0.64 to −0.06; *P* = .02), HGF (β = −0.46; 95% CI, −0.69 to −0.25; *P* < .001), and interferon γ–inducible protein 10 (β = −0.32; 95% CI, −0.62 to −0.10; *P* = .007). Nadir testosterone (ie, day 3) concentrations were inversely associated with concentrations of IL-6 (β = −0.55; 95% CI, −0.87 to −0.31; *P* < .001), IL-1ra (β = −0.39; 95% CI, −1.04, to −0.15; *P* = .009), IL- 2 receptor (β = −0.53; 95% CI, −1.14 to −0.42; *P* < .001), HGF (β = −0.54; 95% CI, −0.92 to −0.30; *P* < .001), MCP-1 (β = −0.46; 95% CI, −1.20 to −0.30; *P* = .002), and monokine induced by γ interferon (β = −0.41; 95% CI, −1.13 to −0.20; *P* = .006).

Estradiol concentrations were positively associated with some cytokine concentrations, and IGF-1 concentrations were negatively associated with some cytokine concentrations, but none of those associations met significance after adjusting for multiple testing and covariates. However, estradiol to testosterone concentration ratios at day 0 were positively associated with concentrations of IL-6 (β = 0.55; 95% CI, 0.19-0.65; *P* < .001) and HGF (β = 0.48; 95% CI, 0.20-0.81; *P* = .002), while the ratios at day 3 were positively associated with concentrations of IL-6 (β = 0.58; 95% CI, 0.25-0.86; *P* = .001), HGF (β = 0.61; 95% CI, 0.27-0.92; *P* = .001), monokine induced by γ interferon (β = 0.51; 95% CI, 0.21-1.18; *P* = .006), MCP-1 (β = 0.50; 95% CI, 0.24-1.18; *P* = .004), and interferon γ–inducible protein 10 (β = 0.46; 95% CI, 0.15-0.96; *P* = .008) on multivariate linear regression analyses using age, BMI, and CCI score.

### Women

Samples were available from 62 women with COVID-19. All except 3 individuals were hospitalized. There were no statistically significant changes in estradiol, testosterone, or IGF-1 concentrations during hospitalization (eFigure 6, eFigure 7, and eFigure 8 in the Supplement). Women with severe COVID-19, compared with women with milder disease, were older (mean [SD] age, 68 [14] years vs 51 [19] years; *P* < .001) and had more comorbidities (median [IQR] CCI score, 2 [2-4] vs 1 [0-2]; *P* < .001) (Table 1). There were no statistically significant differences in hormone concentrations measured at any day in women with vs without severe COVID-19 after adjustment for age, BMI, CCI score, smoking history, and race. Median (IQR) concentrations on day 0, for example, were 10 (1-21) ng/dL vs 14 (1-24) ng/dL for testosterone, 10 (4-50) pg/mL vs 20 (2-45) pg/mL for estradiol (to convert to picomoles per liter, multiply by 3.671), and 92 (52-128) ng/mL vs 108 (66-139) ng/mL for IGF-1 (to convert to nanomoles per liter, multiply by 0.131) (eTable 4 in the Supplement). Median (IQR) estradiol concentrations were similar when comparing women according to mortality status or ICU admission but were higher at day 0 in women who required artificial ventilation vs those who did not (47 [5-57] pg/mL vs 10 [4-28] pg/mL, *P* = .02) (eTable 5 in the Supplement). Estradiol to testosterone ratio did not differ significantly according to ventilator use, ICU admission, or mortality status. There were no statistically significant differences in testosterone or IGF-1 concentrations among these groups (eTable 6 in the Supplement). Estradiol, testosterone, and IGF-1 concentrations at day 0 and 3 were not correlated with any cytokine concentrations measured in women.

### Gene Expression Analyses in Circulating Mononuclear Cells

**Figure 2.**
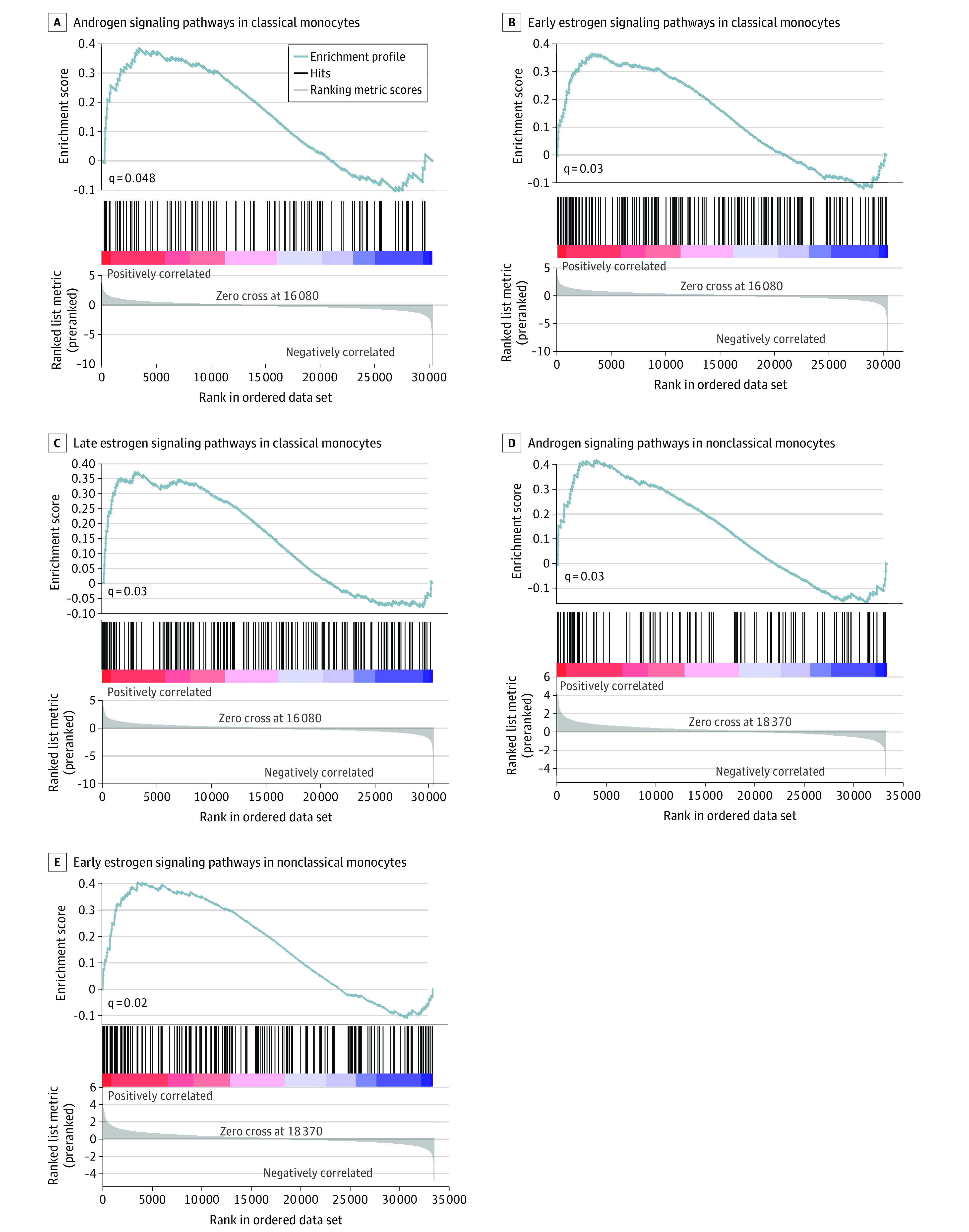
Transcriptional Profiling of Circulating Mononuclear Cells Gene set enrichment analyses were conducted on RNA sequencing data sets from sorted cells based on CD14 and CD16 expression from 7 men with COVID-19 requiring intensive care unit treatment vs 5 men with mild disease. The x axes indicate ranked gene lists (genes are ranked by the sign of the fold change × the −log 10 of the *P* value); colors on the y axes, heat maps of the genes in the gene set (the range of colors [ie, red, pink, light blue, and dark blue] shows the range of the ranking metric [ie, high, moderate, low, and lowest]).

To understand the mechanistic association of altered circulating hormone concentrations with cellular signaling pathways, we accessed RNA sequencing data sets generated from sorted peripheral blood mononuclear cells from patients with severe COVID-19 who required ICU care and those with mild disease who did not require ICU care. These cells were sorted based on surface CD14 or CD16 expression as CD14^+^CD16^−^ (ie, classical) monocytes and CD14^−^CD16^+^ (ie, nonclassical) monocytes. Gene set enrichment analysis revealed hormone signaling pathways among the significantly regulated gene sets (false discovery rate [q] < .05) in both monocyte subsets in men but not women (Figure 2; eTable 7, eTable 8, eTable 9, eTable 10, eTable 11, eTable 12, and eTable 13 in the Supplement). Contrary to the decrease in circulating concentrations of testosterone in patients who needed ICU care vs those who did not, androgen signaling pathways were upregulated in CD14^+^CD16^−^ and CD14^−^CD16^+^ cells in patients requiring ICU care (Figure 2A and 2D). Estrogen signaling pathways were also concomitantly upregulated in patients requiring ICU care, paralleling the increased estrogen to testosterone ratio in this group (Figure 2B, 2C, and 2E).

## Discussion

This cohort study found that men with severe COVID-19 had approximately 65% to 85% lower testosterone concentrations compared with men with a milder disease course, and this difference was independent of other known risk factors associated with severity of COVID-19, such as age, BMI, comorbidities, smoking, and race. Of note, testosterone concentrations were similarly low in men who developed severe COVID-19 illness during their hospital stay as in those who presented with severe illness compared with men with milder courses of COVID-19. In that regard, testosterone was a marker associated with severe and impending severe COVID-19 illness. Epidemiologic data^3^ indicate that while men are not more predisposed to contracting COVID-19, they are more likely to develop severe illness following the infection compared with women. Our study results suggest that, unlike the common presumption, testosterone may not be a propagator of COVID-19 severity in either gender. On the contrary, it may be protective in men.

Testosterone concentrations among men with milder disease course were still lower than the reference range. Indeed, approximately 89% of men at admission demonstrated testosterone concentrations less than the reference range. It is well known that an abrupt change in physical health is associated with an acute suppression of hypothalamic pituitary gonadal axis. Serum testosterone concentrations fall by approximately 50% within 24 hours of an elective surgery, traumatic brain injury, or myocardial infarction^29^ and are inversely associated with severity of illness in patients admitted to the ICU.^30,31^ A suppressive effect on the gonadal axis via inflammatory mediators,^32,33^ decreased testicular responsiveness to gonadotropins, and increased metabolic clearance rate of testosterone have been described as potential causes of lower testosterone concentrations during acute illness.^34,35,36^ We observed a strong inverse association of testosterone concentrations with concentrations of many cytokines, mimicking prior observations in the outpatient setting in other inflammatory states.^37^ It is likely that inflammatory cytokines mediated, at least partly, the association of testosterone with COVID-19 outcomes in our study.

Our study could not determine whether testosterone was a marker or a mediator associated with COVID-19 severity. We did not know the pre-illness serum testosterone concentrations in our study patients. Because patients who came to the hospital were already symptomatic, it is likely that their admission testosterone concentrations had already declined dramatically compared with their baseline concentrations. Alternatively, it is also possible that the men who developed severe COVID-19 had testosterone concentrations that were chronically less than the reference range, even prior to their illness. Men with chronically low testosterone have decreased muscle mass and strength. This may contribute to decreased lung capacity and ventilator dependence.^38,39,40^ This could be an additional explanation for the association between lower testosterone concentrations and worse hospital outcomes in our study patients. Future studies should investigate whether men with testosterone concentrations below the reference range prior to contracting COVID-19 are more likely to develop severe disease. If true, this would support a mediator role for testosterone and suggest that long-term testosterone treatment has potential to prevent respiratory compromise in illnesses and acute infections that target the respiratory tract.

We found that there was no statistically significant change in estradiol concentrations in patients with COVID-19. Indeed, a potential upregulation of aromatase enzyme in adipose tissue during critical illness, possibly due to inflammatory cytokines,^35,41^ is likely to stimulate a multifold increase in conversion of testosterone to estradiol.^35,36^ Consistent with this, we found that higher estradiol to testosterone ratio was associated with inflammatory cytokine concentration, COVID-19 severity, ventilator use, ICU admission, and mortality.

In contrast to the lower circulating testosterone concentrations, our data on gene enrichment showed an upregulation of androgen (and estrogen) signaling pathways in circulating monocytes in men with severe COVID-19. These data point to the likelihood for adaptive upregulation of these signaling pathways in men, which could result from upregulation of cognate receptors or entrainment of alternative pathways that converge on androgen and estrogen-responsive genes.^42^ The increase in androgen signaling may be an adaptive response to the decrease in serum testosterone concentrations, reflecting a counterbalancing mechanism to preserve androgen signaling in the presence of depleted serum hormone. It is also possible that the increased androgen signaling was an outcome associated with critical illness, per se, and was independent of serum testosterone perturbations. Blockade of this signaling, as is being attempted by androgen receptor blockers in patients with COVID-19 in the Hormonal Intervention for the Treatment in Veterans With COVID-19 Requiring Hospitalization study,^43^ could be counterproductive if the increased androgen signaling is an adaptive and beneficial response to critical illness. Alternatively, the converse may be true if the increase in androgen signaling was maladaptive and harmful. In that case, increased androgen signaling would be undesirable. The SARS-CoV-2 virus binds to angiotensin-converting enzyme 2 (ACE2) receptor and undergoes S protein priming by the type II transmembrane serine protease (TMPRSS2) to enter the cells.^44,45^ While TMPRSS2 is regulated by the androgen receptor,^46^ it is not known whether increased androgen signaling would activate ACE2 or TMPRSS2 function. It has also been noted that men on androgen deprivation therapy for prostate cancer have a lower incidence of COVID-19 as compared with matched individuals in control groups.^47,48^ However, impaired mobility associated with sarcopenia induced by androgen deprivation therapy may have decreased their risk of exposure to SARS-CoV-2 virus. Other studies^49,50^ have not confirmed an association between androgen deprivation therapy and SARS-CoV-2 infection or COVID-19 illness. We also found that estrogen signaling was increased in men in the setting of an increased estradiol to testosterone ratio in men with COVID-19. Further research is needed to delineate the role of hormone signaling in acute illnesses.

This study has several strengths. We assessed serial testosterone and estradiol concentrations during the course of hospitalization due to COVID-19. Prior studies^14,15^ have measured sex hormones only at admission to the hospital. Our approach enabled us to examine the association of serum testosterone at presentation to the health care system, as well as that of nadir testosterone concentration, with hospital outcomes. Importantly, to our knowledge, this is the only study to measure sex hormone concentrations in patients in the hospital using liquid chromatography–mass spectrometry. Immunoassays lose their accuracy when hormones circulate at low concentrations^51^ and are not recommended for measurement of testosterone in men who are hypogonadal or for measurement of estradiol in men or in women who are postmenopausal.

### Limitations

Our study also has many limitations. This is an observational study that evaluated associations of sex hormones and IGF-1 with COVID-19. Hence, we could not make interpretations of causality. We did not assess free or bioavailable testosterone concentrations. However, given the 3-fold to 4-fold difference in total testosterone concentrations between men with and without severe COVID-19, it is extremely likely that free testosterone concentrations would also be lower in men with severe COVID-19. Additionally, sex hormone–binding globulin concentrations are increased in acute illnesses, which would further lower the free hormone concentrations in men with severe COVID-19.^22^

## Conclusions

This single center cohort study of patients with COVID-19 found that lower testosterone concentrations and increased estradiol to testosterone ratio during hospitalization were associated with disease severity, inflammation, and mortality in men with COVID-19. These data suggest caution should be practiced with approaches that antagonize testosterone signaling or supplement estrogen to treat men with severe COVID-19.

## Notes

Supplement. **eAppendix.** Supplemental Methods **eTable 1.** Baseline Characteristics, Hormone Concentrations, and Hospital Outcomes in Men and Women **eTable 2.** Serum Estradiol Concentration by Intensive Care Unit Admission, Ventilator Use, and Mortality in Men **eTable 3.** Serum Insulinlike Growth Factor 1 Concentration by Intensive Care Unit Admission, Ventilator Use, and Mortality in Men **eTable 4.** Serial Hormone Concentration in Women With and Without Severe COVID-19 **eTable 5.** Serum Estradiol Concentration by Intensive Care Unit Admission, Ventilator Use, and Mortality in Women **eTable 6.** Serum Testosterone Concentrations by Intensive Care Unit Admission, Ventilator Usage and Mortality in Women **eTable 7.** Gene Set Enrichment Analyses on Hallmark Gene Sets Demonstrating Pathways That Are Upregulated in 7 Men in Intensive Care Units Compared With 5 Men With Mild Disease in CD14^+^CD16^-^ Peripheral Blood Mononuclear Cells **eTable 8.** Gene Set Enrichment Analyses on Hallmark Gene Sets Demonstrating Pathways That Are Upregulated in 4 Women in Intensive Care Units Compared With 4 Women With Mild Disease in CD14^+^CD16^-^ Peripheral Blood Mononuclear Cells **eTable 9.** Gene Set Enrichment Analyses on Hallmark Gene Sets Demonstrating Pathways That Are Downregulated in 7 Men in Intensive Care Units Compared With 5 Men With Mild Disease in CD14^+^CD16^-^ Peripheral Blood Mononuclear Cells **eTable 10.** Gene Set Enrichment Analyses on Hallmark Gene Sets Demonstrating Pathways That Are Upregulated in 7 Men in Intensive Care Units Compared With 5 Men With Mild Disease in CD14^-^CD16^+^ Peripheral Blood Mononuclear Cells **eTable 11.** Gene Set Enrichment Analyses on Hallmark Gene Sets Demonstrating Pathways That Are Upregulated in 4 Women in Intensive Care Units Compared With 4 Women With Mild Disease in CD14^-^CD16^+^ Peripheral Blood Mononuclear Cells **eTable 12.** Gene Set Enrichment Analyses on Hallmark Gene Sets Demonstrating Pathways That Are Downregulated in 7 Men in Intensive Care Units Compared With 5 Men With Mild Disease in CD14^-^CD16^+^ Peripheral Blood Mononuclear Cells **eTable 13.** Gene Set Enrichment Analyses on Hallmark Gene Sets Demonstrating Pathways That Are Downregulated in 4 Women in Intensive Care Units Compared With 4 Women With Mild Disease in CD14^-^CD16^+^ Peripheral Blood Mononuclear Cells **eFigure 1.** Testosterone Concentrations During Hospital Stay in Male Patients **eFigure 2.** Estradiol Concentrations During Hospital Stay in Male Patients **eFigure 3.** Insulinlike Growth Factor 1 Concentrations During Hospital Stay in Male Patients **eFigure 4.** Regression Curve Demonstrating Probability of COVID-19 Severity as Predicted by Testosterone **eFigure 5.** Regression Curves Demonstrating Probability of Intensive Care Unit Admission, Ventilator Usage, or Mortality as Predicted by Testosterone **eFigure 6.** Estradiol Concentrations in Women at Days 0, 3, and 7 **eFigure 7.** Testosterone Concentrations in Women at Days 0, 3, and 7 **eFigure 8.** Insulinlike Growth Factor 1 Concentrations in Women at Days 0, 3, and 7 eReferences
