# Supplementary material for: Association of Circulating Sex Hormones With Inflammation and Disease Severity in Patients With COVID-19
Source: JAMA Netw Open. 2021 May 25;4(5):e2111398. doi: 10.1001/jamanetworkopen.2021.11398 (PMC8150664; doi:10.1001/jamanetworkopen.2021.11398)
Supplement: Supplement. — eAppendix. Supplemental Methods eTable 1. Baseline Characteristics, Hormone Concentrations, and Hospital Outcomes in Men and Women eTable 2. Serum Estradiol Concentration by Intensive Care Unit Admission, Ventilator Use, and Mortality in Men eTable 3. Serum Insulinlike Growth Factor 1 Concentration by Intensive Care Unit Admission, Ventilator Use, and Mortality in Men eTable 4. Serial Hormone Concentration in Women With and Without Severe COVID-19 eTable 5. Serum Estradiol Concentration by Intensive Care Unit Admission, Ventilator Use, and Mortality in Women eTable 6. Serum Testosterone Concentrations by Intensive Care Unit Admission, Ventilator Usage and Mortality in Women eTable 7. Gene Set Enrichment Analyses on Hallmark Gene Sets Demonstrating Pathways That Are Upregulated in 7 Men in Intensive Care Units Compared With 5 Men With Mild Disease in CD14+CD16- Peripheral Blood Mononuclear Cells eTable 8. Gene Set Enrichment Analyses on Hallmark Gene Sets Demonstrating Pathways That Are Upregulated in 4 Women in Intensive Care Units Compared With 4 Women With Mild Disease in CD14+CD16- Peripheral Blood Mononuclear Cells eTable 9. Gene Set Enrichment Analyses on Hallmark Gene Sets Demonstrating Pathways That Are Downregulated in 7 Men in Intensive Care Units Compared With 5 Men With Mild Disease in CD14+CD16- Peripheral Blood Mononuclear Cells eTable 10. Gene Set Enrichment Analyses on Hallmark Gene Sets Demonstrating Pathways That Are Upregulated in 7 Men in Intensive Care Units Compared With 5 Men With Mild Disease in CD14-CD16+ Peripheral Blood Mononuclear Cells eTable 11. Gene Set Enrichment Analyses on Hallmark Gene Sets Demonstrating Pathways That Are Upregulated in 4 Women in Intensive Care Units Compared With 4 Women With Mild Disease in CD14-CD16+ Peripheral Blood Mononuclear Cells eTable 12. Gene Set Enrichment Analyses on Hallmark Gene Sets Demonstrating Pathways That Are Downregulated in 7 Men in Intensive Care Units Compared With 5 Men With Mild Disease i [file jamanetwopen-e2111398-s001.pdf]

Supplemental Online Content

Dhindsa S, Zhang N, McPhaul MJ, et al. Association of circulating sex hormones with inflammation and disease severity in patients with COVID-19. *JAMA Netw Open*. 2021;4(5):e2111398. doi:10.1001/jamanetworkopen.2021.11398

eAppendix. Supplemental Methods

eTable 1. Baseline Characteristics, Hormone Concentrations, and Hospital Outcomes in Men and Women

eTable 2. Serum Estradiol Concentration by Intensive Care Unit Admission, Ventilator Use, and Mortality in Men

eTable 3. Serum Insulinlike Growth Factor 1 Concentration by Intensive Care Unit Admission, Ventilator Use, and Mortality in Men

eTable 4. Serial Hormone Concentration in Women With and Without Severe COVID-19

eTable 5. Serum Estradiol Concentration by Intensive Care Unit Admission, Ventilator Use, and Mortality in Women

eTable 6. Serum Testosterone Concentrations by Intensive Care Unit Admission, Ventilator Usage and Mortality in Women

eTable 7. Gene Set Enrichment Analyses on Hallmark Gene Sets Demonstrating Pathways That Are Upregulated in 7 Men in Intensive Care Units Compared With 5 Men With Mild Disease in $CD14^{+}CD16^{-}$ Peripheral Blood Mononuclear Cells

eTable 8. Gene Set Enrichment Analyses on Hallmark Gene Sets Demonstrating Pathways That Are Upregulated in 4 Women in Intensive Care Units Compared With 4 Women With Mild Disease in $CD14^{+}CD16^{-}$ Peripheral Blood Mononuclear Cells

eTable 9. Gene Set Enrichment Analyses on Hallmark Gene Sets Demonstrating Pathways That Are Downregulated in 7 Men in Intensive Care Units Compared With 5 Men With Mild Disease in $CD14^{+}CD16^{-}$ Peripheral Blood Mononuclear Cells

eTable 10. Gene Set Enrichment Analyses on Hallmark Gene Sets Demonstrating Pathways That Are Upregulated in 7 Men in Intensive Care Units Compared With 5 Men With Mild Disease in $CD14^{-}CD16^{+}$ Peripheral Blood Mononuclear Cells

eTable 11. Gene Set Enrichment Analyses on Hallmark Gene Sets Demonstrating Pathways That Are Upregulated in 4 Women in Intensive Care Units Compared With 4 Women With Mild Disease in $CD14^{-}CD16^{+}$ Peripheral Blood Mononuclear Cells

eTable 12. Gene Set Enrichment Analyses on Hallmark Gene Sets Demonstrating Pathways That Are Downregulated in 7 Men in Intensive Care Units Compared With 5 Men With Mild Disease in $CD14^{-}CD16^{+}$ Peripheral Blood Mononuclear Cells

© 2021 Dhindsa S et al. *JAMA Network Open.*

eTable 13. Gene Set Enrichment Analyses on Hallmark Gene Sets Demonstrating Pathways That Are Downregulated in 4 Women in Intensive Care Units Compared With 4 Women With Mild Disease in $CD14^{-}CD16^{+}$ Peripheral Blood Mononuclear Cells

eFigure 1. Testosterone Concentrations During Hospital Stay in Male Patients

eFigure 2. Estradiol Concentrations During Hospital Stay in Male Patients

eFigure 3. Insulinlike Growth Factor 1 Concentrations During Hospital Stay in Male Patients

eFigure 4. Regression Curve Demonstrating Probability of COVID-19 Severity as Predicted by Testosterone

eFigure 5. Regression Curves Demonstrating Probability of Intensive Care Unit Admission, Ventilator Usage, or Mortality as Predicted by Testosterone

eFigure 6. Estradiol Concentrations in Women at Days 0, 3, and 7

eFigure 7. Testosterone Concentrations in Women at Days 0, 3, and 7

eFigure 8. Insulinlike Growth Factor 1 Concentrations in Women at Days 0, 3, and 7

eReferences

This supplemental material has been provided by the authors to give readers additional information about their work.

© 2021 Dhindsa S et al. *JAMA Network Open.*

eAppendix. Supplementary Methods

We utilized serum samples that were prospectively collected from patients who presented to the Barnes Jewish Hospital in Saint Louis, MO with symptoms suggestive of COVID-19 illness and were confirmed to have SARS-CoV-2 infection with nasopharyngeal swabs. Testosterone, estradiol, and IGF-1 were measured by liquid chromatography- mass spectrometry at the time of presentation to the hospital (baseline or day 0) and at days 3, 7, 14 and 28 after admission (if the patient remained hospitalized).

Hormone assays:

Owing to limited sample volumes available, assays for total testosterone, estradiol, and IGF-1 were performed following dilution into charcoal stripped serum as follows:

Testosterone (females): 1 part sample to 2 parts stripped serum

Testosterone (males): 1 part sample to 3 parts stripped serum

IGF-1 (both sexes): 1 part sample to 1 part stripped serum

Estradiol (both sexes): 1 part sample to 1 part stripped serum

Validation experiments have documented that such dilutions yield reproducible, accurate measurements compared to undiluted specimens (Quest Diagnostics Inc., unpublished information).

Testosterone

Total testosterone was measured by liquid chromatography tandem mass spectrometry (LC-MS/MS)[1]. The method is linear from 205 to 2000 ng/dL with an analytical sensitivity (Limit of quantfication) set at a coefficient of variation (CV) of ≤20%, is 1.0 ng/dl. Intra-assay and total CVs across four quality control concentrations were less than 10%. The reference intervals for

© 2021 Dhindsa S et al. *JAMA Network Open.*

total testosterone for this assay were 250-1100 ng/dL in adult males [2] and 2-45 ng/dl in adult females. The performing laboratory is certified by Clinical Laboratory Improvement Amendments and is a participant in the Hormone Standardization program of the Centers for Disease Control and Prevention.

Estradiol

LC-MS/MS methodology was also used to measure total estradiol. The method was similar to that previously described [3], with some improvements After adding 300 µL of ethanol spiked with internal standards (17β-Estradiol-2,3,4-$^{13}C_3$) to 200 µL of patient serum, the mixture was incubated in an ice slurry for 30 minutes before centrifugation. 50 µL of the supernatant was injected onto a multiplex High Performance Liquid Chromatography (HPLC) system equipped with an online turbo flow extraction system (Cohesive Technologies Inc.; Franklin, MA, part of Thermo Fisher Scientific) for further clean up and separation from the matrix. The online extraction and HPLC columns were Phenomenex Strata C18-E (70 um, 50x1.0 mm; P/N 00B-S001-A0) and Phenomenex Biphenyl (2.6 um, 100Å, 50x3.0 mm; P/N AF0-5727) respectively. Estradiol and its internal standard were detected by a triple quadrupole mass spectrometer (Sciex 6500+ Framingham, MA) using H-ESI source and operating in Multiple Reaction Monitoring (MRM) mode. The MRM transitions were (m/z) 271.17 to 143.05 and 145.05 for Estradiol and 274.18 to 148.00 and 186.05 for the Internal Standard. The ratio of the analyte response relative to that of the internal standard were used to build a calibration curve and to quantify Estradiol in patient samples. The Inter assay precision (CV) of the assay is 4.06% at 20 pg/mL of, 1.42% at 200 pg/mL, and 1.88% at 800 pg/mL. The LOQ for estradiol in this assay is 2 pg/mL. The

reference range is ≤29 pg/mL for men, 39-440 pg/mL for premenopausal women and ≤10 pg/mL for postmenopausal women.

IGF-1 measurements

IGF-1 was measured by highly accurate LC-MS method similar to the method described previously.[4] 100 µL aliquots of patient sera were mixed with 10 µL of internal standard (15N-labeled IGF-1, ProSpec) and 400 µL of acidified ethanol solution (87.5% EtOH, 12.5% 1 N HCl) and mixed vigorously. After incubating at room temperature for 30 min, and centrifugation, 350 µL of supernatant was mixed with 60 µL of 1.5 M Tris base. The samples were cooled for 60 min at -20°C and centrifuged again to remove additional protein precipitate. The IGF-1 and its internal standard were separated further from matrix using a multiplex high performance liquid chromatography (HPLC) system (Aria TLX-4) equipped with an on-line extraction (Thermo Scientific, San Jose, CA). The extraction column was a Phenomenex Monolithic Onyx C18 Guard Cartridge; the analytical column was a Phenomenex Kinetex C18 50 × 4.6 mm, 100 Å pore size. A Thermo Scientific Q Exactive Focus Hybrid Quadrupole-Orbitrap mass spectrometer (Thermo Scientific, San Jose, CA) was used as the detection system equipped with heated electrospray ionization (HESI) source and operating in positive All Ion Fragmentation (AIF) mode. After IGF-1 quantitation, the chromatograms were then examined for the presence of variants. No variant was found in this study.

The inter assay precision (CV) of the IGF-1 assay is 8.79% at 40 ng/mL, 6.43% at 177 ng/mL, and 5.97% at 358 ng/mL. The LOQ for IGF-1 assay is 8 ng/mL. The reference ranges of IGF-1 vary by age and gender and have been described previously[5].

© 2021 Dhindsa S et al. *JAMA Network Open.*

Cytokine Quantification: Plasma obtained from subjects was frozen at -80°C and subsequently analyzed using a human magnetic cytokine panel providing parallel measurement of 35 cytokines (ThermoFisher). These are Interleukin (IL)-1α, 1β, 2-10, 12, 13, 15, 17A, 17F and 22, IL-1 receptor antagonist (IL-1RA), IL-2R, Fibroblast growth factor (FGF)-basic, Tumor necrosis factor (TNF)-α, Granulocyte colony-stimulating factor (G-CSF) and Granulocyte-macrophage colony-stimulating factor (GM-CSF), RANTES (Regulated on Activation, Normal T Cell Expressed and Secreted), Eotaxin, Macrophage Inflammatory Proteins (MIP)-1α and 1β, monocyte chemoattractant protein-1 (MCP-1), Epidermal growth factor (EGF), hepatocyte growth factor (HGF), Vascular endothelial growth factor (VEGF), Interferon (IFN)-α and γ, interferon-γ-inducible protein 10 (IP-10) and monokine induced by gamma interferon (MIG) . The assay was performed according to the manufacturer's instructions with each subject sample performed in duplicate and then analyzed on a Luminex FLEXMAP 3D instrument.

PBMC sorting and RNA sequencing

Cryopreserved peripheral blood mononuclear cells (PBMCs) from COVID patients were thawed and washed with HBSS with 2mM EDTA and 0.04% LPS-free BSA. These PBMCs were then blocked with 5% goat serum and stained on ice with anti-CD14-APC/Cy7, anti-CD16-PE/Cy7, anti-CD15 PerCP/Cy5.5, and anti-CD56-Alexa Fluor 700 for 30 minutes and re-suspended in the aforementioned HBSS buffer. Dead cells were excluded based on positive Helix NP™ Green staining. The cells were then sorted straight into Trizol by FACS Aria II. Total RNA was isolated as per Trizol protocol. RNA of each sample was sequenced at the Genome Technology Access Center of the McDonnell Genome Institute at Washington University in St. Louis. Total RNA integrity was determined using Agilent Bioanalyzer or 4200 Tapestation. Library preparation

© 2021 Dhindsa S et al. *JAMA Network Open.*

was performed with 10ng of total RNA with a Bioanalyzer RIN score greater than 8.0. ds-cDNA was prepared using the SMARTer Ultra Low RNA kit for Illumina Sequencing (Takara-Clontech) per manufacturer's protocol. cDNA was fragmented using a Covaris E220 sonicator using peak incident power 18, duty factor 20%, cycles per burst 50 for 120 seconds. cDNA was blunt ended, had an A base added to the 3' ends, and then had Illumina sequencing adapters ligated to the ends. Ligated fragments were then amplified for 12-15 cycles using primers incorporating unique dual index tags. Fragments were sequenced on an Illumina NovaSeq-6000 using paired end reads extending 150 bases at the Genome Technology Access Center of the McDonnell Genome Institute at Washington University in St. Louis.

Basecalls and demultiplexing were performed with Illumina's bcl2fastq software and a custom python demultiplexing program with a maximum of one mismatch in the indexing read. RNA-seq reads were then aligned to the Ensembl release 76 top-level assembly of the human genome (GRCh38) along with the SARS-CoV-2 reference genome with STAR version 2.0.4b[6] with the parameters --outStd BAM_Unsorted --outMultimapperOrder Random --outSAMtype BAM Unsorted --outSAMunmapped None --outSAMmapqUnique 60 --outFilterScoreMinOverLread 0 --outFilterMatchNmin 20 --outFilterMatchNminOverLread 0 --outFilterMismatchNoverLmax 0.1 --clip3pAdapterSeq AGATCGGAAGAGCGGTTCAGCAGGAATGCCGAG AGATCGGAAGAGCGTCGTGTAGGGAAAGAGTGTA. Gene counts were derived from the number of uniquely aligned unambiguous reads by Subread:featureCount version 1.4.5[7] to Ensembl and SARS-CoV-2 gene models. Sequencing performance was assessed for the total number of aligned reads, total number of uniquely aligned reads, and features detected. Differential expression analysis to determine genes that differed with severe disease for each cell

type and both male and females was performed using DESeq2 (v1.24)[8]. Genes without at least 10 read counts across all samples were removed prior to analysis. Gene Set Enrichment Analysis (v4.0.3)[9] was performed using the GSEAPreranked tool against the Hallmark gene set (v7.2). Genes were ranked using the sign of the fold change times the -log10(p-value) and otherwise default parameters.

Statistical analysis

We assessed the relation of testosterone, estradiol, and IGF-1 concentrations during the hospitalization with COVID severity, ICU and ventilator use, mortality and inflammatory cytokines using univariate and multivariate regression and mixed model analyses. These models addressed missing data values by maximum likelihood, under the data missing at random assumption. Sensitivity analyses were performed to evaluate this assumption. Based on these analyses, missing at random assumption was deemed reasonable. The comparisons were adjusted for age, BMI, race, smoking and co-morbidities at baseline (Charlson Comorbidity Index, CCI). CCI is a validated method to determine significant medical comorbidity. It considers 17 common medical conditions that predict one-year mortality. These include myocardial infarction, heart failure, peripheral arterial disease, cerebrovascular disease, dementia, chronic pulmonary disease, connective tissue disease, ulcer disease, liver disease, diabetes (including diabetes with end organ damage), hemiplegia, renal disease, leukemia, lymphoma, solid tumors, and AIDS. Medical diagnoses are weighted for severity and summed to provide a weighted index of medical comorbidity with total scores ranging from 0-33.

© 2021 Dhindsa S et al. *JAMA Network Open.*

eTable 1. Baseline Characteristics, Hormone Concentrations, and Hospital Outcomes in Men and Women

	Males (N=90)	Females (N=62)	P
Age (years)	64±14	61±18	0.33
BMI (kg/m^2)	27.7±6.8	33±8.8	<0.001
Charlson comorbidity index	3 [1, 4]	2 [1, 3]	0.046
Severe COVID	71%	60%	0.14
ICU	59%	47%	0.14
ventilator	27%	21%	0.42
Mortality	28%	19%	0.23
Testosterone (ng/dl)	79 [38, 181]	12 [1, 21]	<0.001
Estradiol (pg/ml)	15 [10, 22]	15 [3, 47]	0.34
IGF-1 (ng/ml)	90 [62, 126]	106 [62, 127]	0.90
CRP (mg/L)	122 [58, 214]	96 [48, 174]	0.71

© 2021 Dhindsa S et al. *JAMA Network Open.*

eTable 2. Serum Estradiol Concentration by Intensive Care Unit Admission, Ventilator Use, and Mortality in Men

		Day 0	Day 3	Day 7	Day 14	Day 28
ICU	Yes (N=53)	15 [11, 25]	13 [7, 26]	13 [7, 20]	18 [10, 23]	13 [10, 26]
	No (N=37)	13 [10, 20]	13 [9, 26]	12 [10, 13]	No data	15 [9, 20]
Ventilator usage	Yes (N=24)	22 [14, 31]	10 [6, 31]	11 [4, 21]	19 [12, 23]	13 [12, 33]
	No (N= 66)	13 [10, 19]	13 [9, 19]	13 [11, 21]	9 [8, 17]	13 [12, 33]
Mortality	Deceased (N= 25)	18 [12, 27]	16 [9, 33]	16 [2, 30]	15 [9, 23]	No data
	Alive (65)	14 [10, 21]	12 [7, 19]	12 [9, 15]	19 [9, 22]	13 [10, 20]

© 2021 Dhindsa S et al. *JAMA Network Open.*

eTable 3. Serum Insulinlike Growth Factor 1 Concentration by Intensive Care Unit Admission, Ventilator Use, and Mortality in Men

		Day 0	Day 3	Day 7	Day 14	Day 28
ICU	Yes (N=53)	87 [57, 117]	78 [51, 116]	72 [41, 111]	110 [41, 124]	No data
	No (N=37)	95 [63, 149]	75 [16, 136]	87 [54, 135]	No data	No data
Ventilator usage	Yes (N=24)	77 [53, 113]	88 [44, 115]	75 [40, 138]	112 [41, 131]	No data
	No (N= 66)	90 [63, 134]	74 [39, 123]	79 [55, 110]	101 [42, 173]	No data
Mortality	Deceased (N= 25)	78 [59, 98]	65 [40, 99]	69 [53, 119]	No data	No data
	Alive (65)	98 [62, 137]	85 [46, 120]	82 [40, 117]	122 [79, 151]	79 [58, 147]

eTable 4. Serial Hormone Concentration in Women With and Without Severe COVID-19

	Severe COVID	Day 0	Day 3	Day 7
Testosterone (ng/dl)	Yes	10 [1, 21]	9 [3, 32]	15 [5, 24]
	No	14 [1, 24]	10 [5, 13]	No data
Estradiol (pg/ml)	Yes	10 [4, 50]	20 [10, 31]	16 [8, 47]
	No	20 [2, 45]	13 [8, 42]	No data
IGF-1 (ng/ml)	Yes	92 [52, 128]	70 [40, 91]	65 [43, 105]
	No	108 [66, 139]	87 [55, 151]	No data
Estradiol/Testosterone (%)	Yes	16 [6, 58]	23 [11, 120]	19 [5, 16]
	No	29 [21, 40]	20 [18, 80]	No data

#p<0.05 for comparison with day 0

"No data" is indicated if there were insufficient patients in that category.

© 2021 Dhindsa S et al. *JAMA Network Open.*

eTable 5. Serum Estradiol Concentration by Intensive Care Unit Admission, Ventilator Use, and Mortality in Women

		Day 0	Day 3	Day 7
ICU	Yes (N=29)	10 [2, 48]	20 [11, 30]	16 [8, 52]
	No (N=33)	20 [5, 45]	16 [10, 38]	29 [17, 34]
ventilator	Yes (N=13)	47 [5, 57]*	30 [23, 68]#	16 [10, 103]
	No (N=49)	10 [4, 28]	14 [9, 25]	21 [8, 32]
Mortality	Deceased (N=12)	8 [2, 56]	22 [15, 24]	16 [11, 108]
	Alive (N=50)	16 [5, 45]	15 [9, 35]	21 [8, 32]

*p=0.02 as compared to women without ventilator use, adjusted for group differences in age, BMI, CCI, smoking status and race.

#p<0.05 for comparison with day 0

© 2021 Dhindsa S et al. *JAMA Network Open.*

eTable 6: Serum Testosterone Concentrations by Intensive Care Unit Admission, Ventilator Usage and Mortality in Women

		Day 0	Day 3	Day 7
ICU	Yes (N=29)	10 [4, 21]	8 [3, 22]	15 [4, 24]
	No (N=33)	14 [1, 23]	10 [5, 16]	14 [11, 20]
ventilator	Yes (N=13)	19 [8, 39]	29 [8, 47]	18 [8, 35]
	No (N=49)	10 [1, 20]	7 [3, 14]	14 [8, 17]
Mortality	Deceased (N=12)	15 [4, 39]	9 [1, 45]	35 [4, 39]
	Alive (N=50)	11 [1, 20]	10 [4, 17]	14 [8, 17]

*p<0.05 as compared to women with comparator group (no ICU stay, no ventilator use or alive), adjusted for group differences in age, BMI, smoking status and race.

© 2021 Dhindsa S et al. *JAMA Network Open.*

eTable 7.: Gene Set Enrichment Analyses on Hallmark Gene Sets Demonstrating Pathways That Are Upregulated in 7 Men in Intensive Care Units Compared With 5 Men With Mild Disease in $CD14^{+}CD16^{-}$ Peripheral Blood Mononuclear Cells

GS follow link to MSigDB	GS DETAILS	SIZE	ES	NES	NOM p-val	FDR q-val	FWER p-val	RANK AT MAX	LEADING EDGE	
1	HALLMARK_TNFA_SIGNALING_VIA_NFKB	Details …	193	0.59	2.35	0	0	0	3023	tags=35%, list=10%, signal=39%
2	HALLMARK_INFLAMMATORY_RESPONSE	Details …	185	0.51	2.04	0	0.001	0.001	4090	tags=32%, list=13%, signal=37%
3	HALLMARK_UV_RESPONSE_DN	Details …	138	0.51	1.93	0	0.001	0.003	6229	tags=36%, list=21%, signal=45%
4	HALLMARK_IL2_STAT5_SIGNALING	Details …	189	0.48	1.9	0	0.001	0.003	3004	tags=26%, list=10%, signal=29%
5	HALLMARK_REACTIVE_OXYGEN_SPECIES_PATHWAY	Details …	48	0.58	1.86	0	0.001	0.004	4295	tags=38%, list=14%, signal=44%
6	HALLMARK_HYPOXIA	Details …	184	0.47	1.85	0	0.001	0.005	3226	tags=25%, list=11%, signal=28%

© 2021 Dhindsa S et al. *JAMA Network Open.*

7	HALLMARK_OXIDATIVE_PHOSPHORYLATION	Details ...	199	0.45	1.8	0	0.002	0.009	5685	tags=38%, list=19%, signal=46%
8	HALLMARK_COAGULATION	Details ...	115	0.48	1.8	0	0.002	0.01	6073	tags=40%, list=20%, signal=50%
9	HALLMARK_ANGIOGENESIS	Details ...	28	0.61	1.72	0.005	0.003	0.024	3456	tags=32%, list=11%, signal=36%
10	HALLMARK_XENOBIOTIC_METABOLISM	Details ...	183	0.43	1.7	0	0.003	0.026	3941	tags=25%, list=13%, signal=28%
11	HALLMARK_KRAS_SIGNALING_UP	Details ...	178	0.44	1.7	0	0.003	0.027	2937	tags=20%, list=10%, signal=22%
12	HALLMARK_TGF_BETA_SIGNALING	Details ...	52	0.53	1.69	0.004	0.003	0.031	3273	tags=35%, list=11%, signal=39%
13	HALLMARK_MTORC1_SIGNALING	Details ...	200	0.41	1.64	0	0.006	0.061	6342	tags=34%, list=21%, signal=43%
14	HALLMARK_EPITHELIAL_MESENCHYMAL_TRANSITION	Details ...	168	0.41	1.6	0	0.008	0.087	6860	tags=37%, list=23%, signal=47%
15	HALLMARK_COMPLEMENT	Details ...	189	0.4	1.58	0	0.01	0.107	5096	tags=29%, list=17%, signal=34%

© 2021 Dhindsa S et al. *JAMA Network Open.*

16	HALLMARK_HEME_METABOLISM	Details ...	188	0.4	1.57	0	0.01	0.118	3315	tags=21%, list=11%, signal=24%
17	HALLMARK_INTERFERON_GAMMA_RESPONSE	Details ...	199	0.39	1.56	0	0.011	0.133	4176	tags=22%, list=14%, signal=25%
18	HALLMARK_ADIPOGENESIS	Details ...	195	0.37	1.47	0.003	0.026	0.31	6086	tags=33%, list=20%, signal=41%
19	HALLMARK_ESTROGEN_RESPONSE_EARLY	Details ...	179	0.37	1.46	0.009	0.028	0.339	3231	tags=19%, list=11%, signal=21%
20	HALLMARK_ESTROGEN_RESPONSE_LATE	Details ...	177	0.37	1.45	0.003	0.028	0.366	3167	tags=17%, list=10%, signal=19%
21	HALLMARK_WNT_BETA_CATENIN_SIGNALING	Details ...	35	0.46	1.4	0.064	0.045	0.529	5956	tags=43%, list=20%, signal=53%
22	HALLMARK_ANDROGEN_RESPONSE	Details ...	98	0.38	1.39	0.038	0.048	0.572	3376	tags=20%, list=11%, signal=23%

Pathways shown are based on cutoffs defined as q<0.05

© 2021 Dhindsa S et al. *JAMA Network Open.*

eTable 8. Gene Set Enrichment Analyses on Hallmark Gene Sets Demonstrating Pathways That Are Upregulated in 4 Women in Intensive Care Units Compared With 4 Women With Mild Disease in $CD14^{+}CD16^{-}$ Peripheral Blood Mononuclear Cells

GS DETAILS	SIZE	ES	NES	NOM p-val	FDR q-val	FWER p-val	RANK AT MAX	LEADING EDGE	
HALLMARK_EPITHELIAL_MESENCHYMAL_TRANSITION	Details ...	179	0.46	1.69	0	0.025	0.025	7040	tags=37%, list=21%, signal=46%
HALLMARK_COAGULATION	Details ...	128	0.48	1.68	0.001	0.013	0.026	7771	tags=39%, list=23%, signal=51%
HALLMARK_PI3K_AKT_MTOR_SIGNALING	Details ...	97	0.47	1.62	0.001	0.019	0.056	7260	tags=36%, list=22%, signal=46%
HALLMARK_ANGIOGENESIS	Details ...	32	0.55	1.55	0.02	0.041	0.157	3652	tags=31%, list=11%, signal=35%

Pathways shown are based on cutoffs defined as q<0.05

© 2021 Dhindsa S et al. *JAMA Network Open.*

eTable 9. Gene Set Enrichment Analyses on Hallmark Gene Sets Demonstrating Pathways That Are Downregulated in 7 Men in Intensive Care Units Compared With 5 Men With Mild Disease in $CD14^{+}CD16^{-}$ Peripheral Blood Mononuclear Cells

GS DETAILS	SIZE	ES	NES	NOM p-val	FDR q-val	FWER p-val	RANK AT MAX	LEADING EDGE	
HALLMARK_MYC_TARGETS_V2	Details ...	58	-0.49	-1.68	0	0.015	0.028	5629	tags=45%, list=19%, signal=55%
HALLMARK_E2F_TARGETS	Details ...	199	-0.39	-1.63	0	0.011	0.043	4401	tags=32%, list=15%, signal=37%

Pathways shown are based on cutoffs defined by q<0.05. No pathways were observed to be significantly downregulated in Female ICU patients as compared to those with mild disease in in CD14+CD16- peripheral blood mononuclear cells.

© 2021 Dhindsa S et al. *JAMA Network Open.*

eTable 10. Gene Set Enrichment Analyses on Hallmark Gene Sets Demonstrating Pathways That Are Upregulated in 7 Men in Intensive Care Units Compared With 5 Men With Mild Disease in $CD14^{-}CD16^{+}$ Peripheral Blood Mononuclear Cells

	GS<br>follow link to MSigDB	GS DETAILS	SIZE	ES	NES	NOM p-val	FDR q-val	FWER p-val	RANK AT MAX	LEADING EDGE
1	HALLMARK_TNFA_SIGNALING_VIA_NFKB	Details ...	194	0.59	2.17	0	0	0	4007	tags=34%, list=12%, signal=38%
2	HALLMARK_CHOLESTEROL_HOMEOSTASIS	Details ...	72	0.57	1.9	0	0	0	3798	tags=36%, list=11%, signal=41%
3	HALLMARK_TGF_BETA_SIGNALING	Details ...	51	0.58	1.8	0	0.002	0.005	6166	tags=41%, list=18%, signal=50%
4	HALLMARK_IL2_STAT5_SIGNALING	Details ...	193	0.49	1.79	0	0.001	0.005	3835	tags=25%, list=11%, signal=28%
5	HALLMARK_MYOGENESIS	Details ...	178	0.49	1.78	0	0.001	0.005	3496	tags=23%, list=10%, signal=26%
6	HALLMARK_COAGULATION	Details ...	127	0.49	1.73	0	0.002	0.013	9258	tags=43%, list=28%, signal=60%
7	HALLMARK_UV_RESPONSE_DN	Details ...	138	0.47	1.68	0.001	0.004	0.025	5708	tags=30%, list=17%,

© 2021 Dhindsa S et al. *JAMA Network Open.*

										signal=36%
8	HALLMARK_HYPOXIA	Details ...	186	0.46	1.68	0	0.003	0.026	3420	tags=22%, list=10%, signal=24%
9	HALLMARK_INFLAMMATORY_RESPONSE	Details ...	192	0.44	1.63	0	0.006	0.055	6182	tags=29%, list=19%, signal=36%
10	HALLMARK_ANGIOGENESIS	Details ...	30	0.57	1.59	0.012	0.01	0.093	4986	tags=37%, list=15%, signal=43%
11	HALLMARK_REACTIVE_OXYGEN_SPECIES_PATHWAY	Details ...	49	0.5	1.56	0.015	0.013	0.133	4464	tags=31%, list=13%, signal=35%
12	HALLMARK_XENOBIOTIC_METABOLISM	Details ...	187	0.42	1.54	0	0.014	0.163	6013	tags=26%, list=18%, signal=32%
13	HALLMARK_KRAS_SIGNALING_UP	Details ...	184	0.42	1.52	0.003	0.018	0.21	4200	tags=20%, list=13%, signal=23%
14	HALLMARK_COMPLEMENT	Details ...	195	0.41	1.52	0.001	0.017	0.219	6048	tags=30%, list=18%, signal=36%
15	HALLMARK_EPITHELIAL_MESENCHYMAL_TRANSITION	Details ...	178	0.41	1.49	0.003	0.021	0.275	6487	tags=28%, list=19%, signal=34%

© 2021 Dhindsa S et al. *JAMA Network Open.*

16	HALLMARK_ESTROGEN_RESPONSE_EARLY	Details ...	189	0.41	1.49	0	0.021	0.29	3609	tags=22%, list=11%, signal=24%
17	HALLMARK_IL6_JAK_STAT3_SIGNALING	Details ...	83	0.44	1.49	0.016	0.02	0.295	3311	tags=23%, list=10%, signal=25%
18	HALLMARK_ANDROGEN_RESPONSE	Details ...	99	0.42	1.46	0.017	0.03	0.428	4057	tags=25%, list=12%, signal=29%
19	HALLMARK_WNT_BETA_CATENIN_SIGNALING	Details ...	38	0.5	1.44	0.046	0.034	0.488	4427	tags=26%, list=13%, signal=30%

Pathways shown are based on cutoffs defined by q<0.05

© 2021 Dhindsa S et al. *JAMA Network Open.*

eTable 11. Gene Set Enrichment Analyses on Hallmark Gene Sets Demonstrating Pathways That Are Upregulated in 4 Women in Intensive Care Units Compared With 4 Women With Mild Disease in $CD14^{-}CD16^{+}$ Peripheral Blood Mononuclear Cells

	GS DETAILS follow link to MSigDB	SIZE	ES	NES	NOM p-val	FDR q-val	FWER p-val	RANK AT MAX	LEADING EDGE	
1	HALLMARK_ANGIOGENESIS	Details ...	30	0.68	1.92	0.002	0.002	0.002	1610	tags=27%, list=5%, signal=28%
2	HALLMARK_COAGULATION	Details ...	122	0.52	1.86	0	0.002	0.005	5784	tags=35%, list=19%, signal=43%
3	HALLMARK_IL6_JAK_STAT3_SIGNALING	Details ...	83	0.54	1.82	0	0.002	0.007	4873	tags=34%, list=16%, signal=40%
4	HALLMARK_INFLAMMATORY_RESPONSE	Details ...	187	0.46	1.75	0	0.005	0.02	3113	tags=25%, list=10%, signal=28%
5	HALLMARK_EPITHELIAL_MESENCHYMAL_TRANSITION	Details ...	176	0.44	1.66	0	0.009	0.049	3591	tags=22%, list=12%, signal=25%
6	HALLMARK_IL2_STAT5_SIGNALING	Details ...	192	0.42	1.6	0.001	0.019	0.116	3312	tags=20%, list=11%, signal=23%
7	HALLMARK_REACTIVE_OXYGEN_SPECIES_PATHWAY	Details ...	49	0.51	1.59	0.01	0.018	0.123	2649	tags=22%, list=9%, signal=25%

© 2021 Dhindsa S et al. *JAMA Network Open.*

8	HALLMARK_ADIPOGENESIS	Details ...	195	0.41	1.56	0.005	0.022	0.169	8251	tags=44%, list=27%, signal=60%

Pathways shown are based on cutoffs defined as q<0.05

© 2021 Dhindsa S et al. *JAMA Network Open.*

eTable 12. Gene Set Enrichment Analyses on Hallmark Gene Sets Demonstrating Pathways That Are Downregulated in 7 Men in Intensive Care Units Compared With 5 Men With Mild Disease in CD14⁻CD16⁺ Peripheral Blood Mononuclear Cells

GS follow link to MSigDB	GS DETAILS	SIZE	ES	NES	NOM p-val	FDR q-val	FWER p-val	RANK AT MAX	LEADING EDGE	
1	HALLMARK_MYC_TARGETS_V1	Details ...	200	-0.37	-1.67	0	0.019	0.017	4454	tags=29%, list=13%, signal=34%
2	HALLMARK_MYC_TARGETS_V2	Details ...	58	-0.44	-1.64	0.012	0.012	0.023	4074	tags=31%, list=12%, signal=35%
3	HALLMARK_E2F_TARGETS	Details ...	198	-0.33	-1.53	0	0.02	0.056	3927	tags=28%, list=12%, signal=

Pathways shown are based on cutoffs defined as q<0.05

© 2021 Dhindsa S et al. *JAMA Network Open.*

eTable 13. Gene Set Enrichment Analyses on Hallmark Gene Sets Demonstrating Pathways That Are Downregulated in 4 Women in Intensive Care Units Compared With 4 Women With Mild Disease in $CD14^{-}CD16^{+}$ Peripheral Blood Mononuclear Cells

GS DETAILS	SIZE	ES	NES	NOM p-val	FDR q-val	FWER p-val	RANK AT MAX	LEADING EDGE	
HALLMARK_MITOTIC_SPINDLE	Details ...	198	-0.37	-1.6	0	0.072	0.064	5838	tags=35%, list=19%, signal=43%
HALLMARK_E2F_TARGETS	Details ...	198	-0.36	-1.57	0	0.042	0.073	5859	tags=31%, list=19%, signal=38%
HALLMARK_P53_PATHWAY	Details ...	193	-0.37	-1.57	0	0.028	0.073	3916	tags=23%, list=13%, signal=27%
HALLMARK_TGF_BETA_SIGNALING	Details ...	53	-0.44	-1.56	0.018	0.022	0.078	2192	tags=23%, list=7%, signal=24%
HALLMARK_G2M_CHECKPOINT	Details ...	198	-0.3	-1.31	0.026	0.146	0.481	4658	tags=26%, list=15%, signal=31%

Pathways shown are based on cutoffs defined as q<0.05

© 2021 Dhindsa S et al. *JAMA Network Open.*

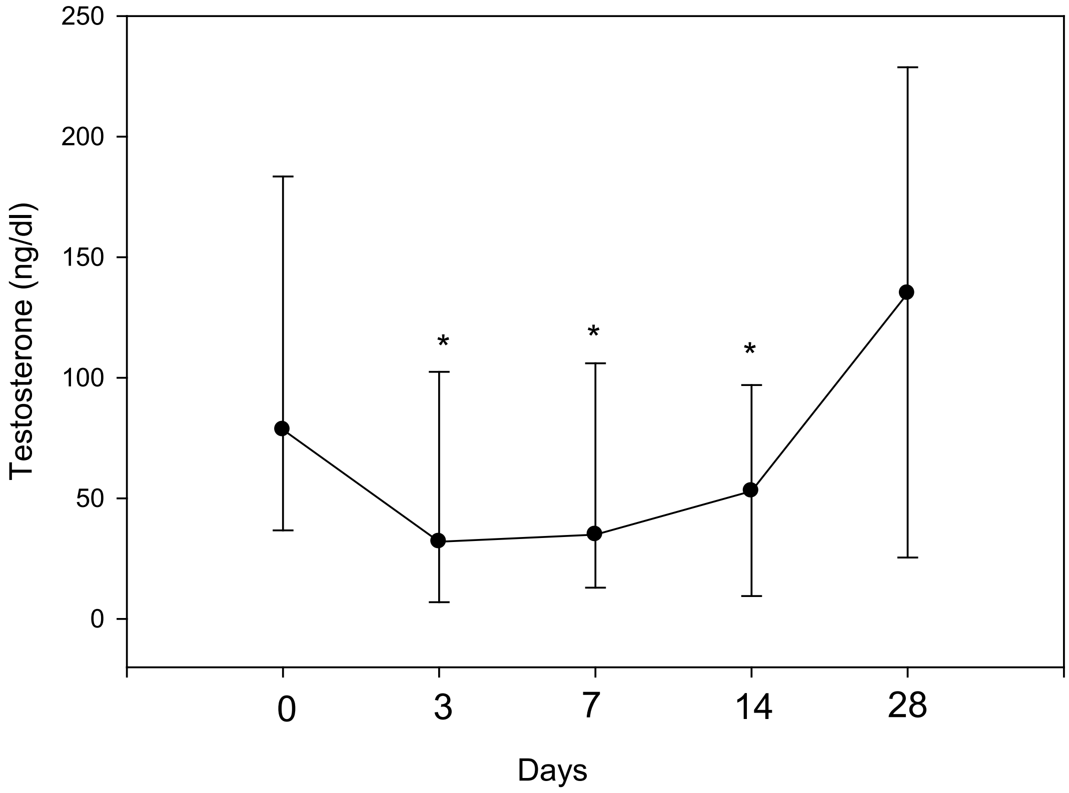

eFigure 1. Testosterone Concentrations During Hospital Stay in Male Patients

Values are median [25th, 75th percentile]. Day 0 includes the 6 patients who were not hospitalized. Serum testosterone concentrations were available on days 0, 3, 7, 14 and 28 in 76, 53, 41, 21 and 12 patients, respectively. * $p<0.05$ as compared to day 0.

© 2021 Dhindsa S et al. *JAMA Network Open.*

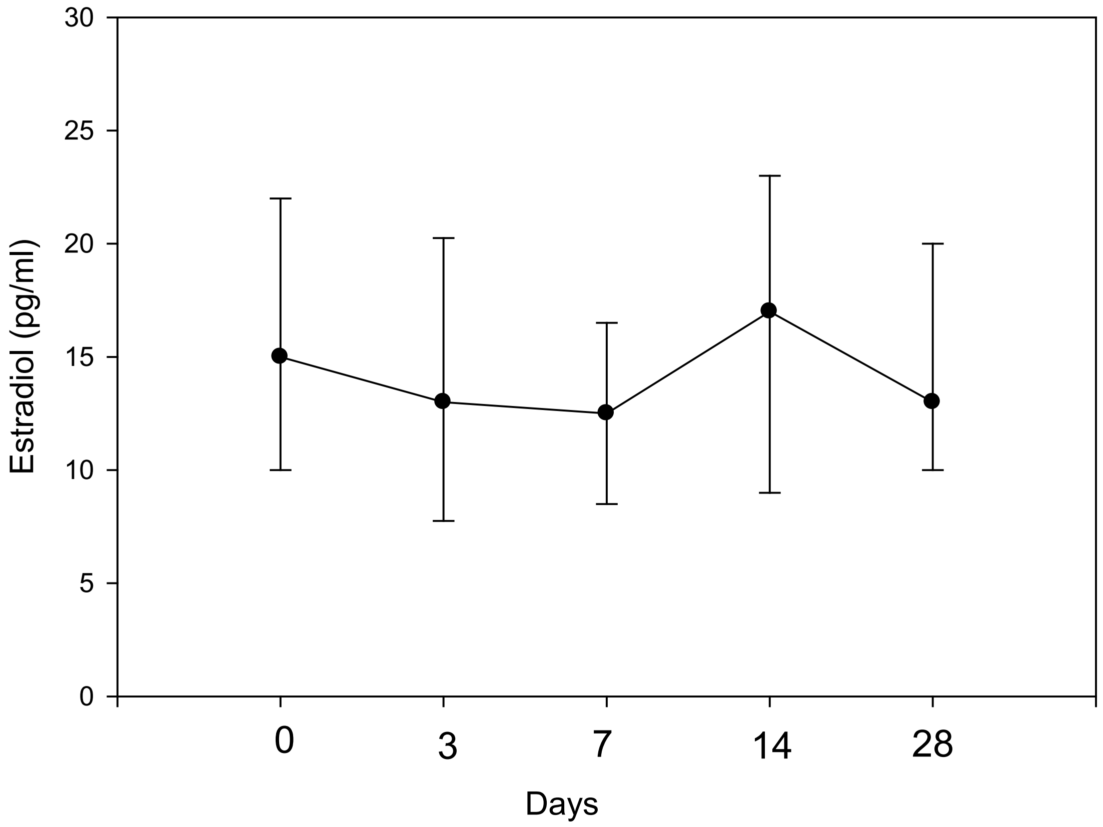

eFigure 2. Estradiol Concentrations During Hospital Stay in Male Patients

Values are median [25th, 75th percentile]. Day 0 includes the 6 patients who were not hospitalized. Serum estradiol concentrations were available on days 0, 3, 7, 14 and 28 in 55, 42, 30, 19 and 11 patients, respectively.

© 2021 Dhindsa S et al. *JAMA Network Open.*

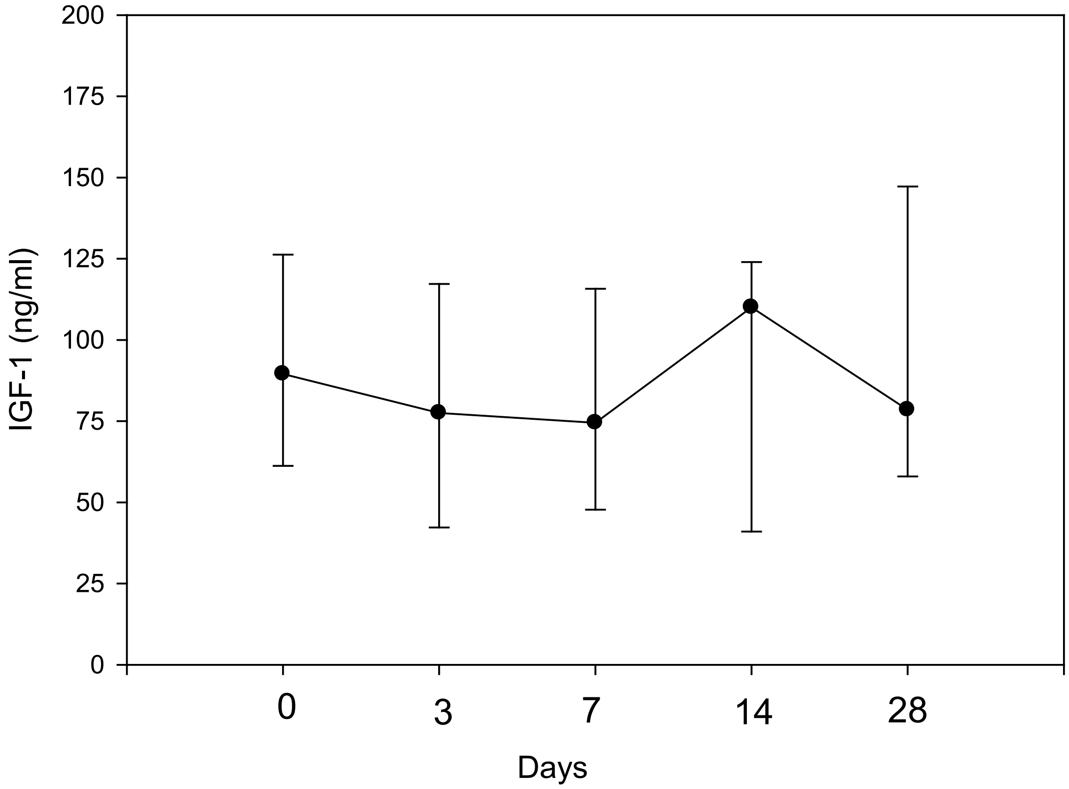

eFigure 3. Insulinlike Growth Factor 1 Concentrations During Hospital Stay in Male Patients

Values are median [25th, 75th percentile]. Day 0 includes the 6 patients who were not hospitalized. Serum IGF-1 concentrations were available on days 0, 3, 7, 14 and 28 in 64, 36, 30, 15 and 10 patients, respectively.

© 2021 Dhindsa S et al. *JAMA Network Open.*

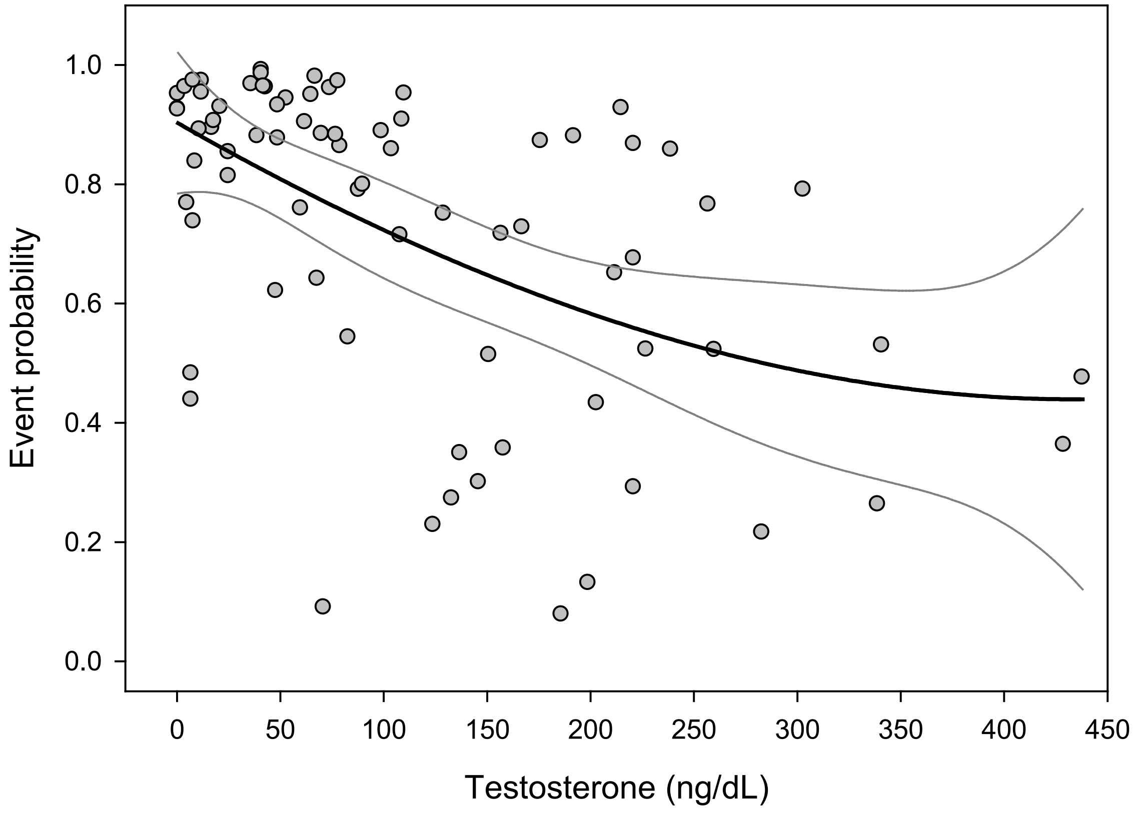

eFigure 4. Regression Curve Demonstrating Probability of COVID-19 Severity as Predicted by Testosterone

Includes 95% confidence intervals. After multivariate adjustment for age, BMI, CCI, smoking and race.

© 2021 Dhindsa S et al. *JAMA Network Open.*

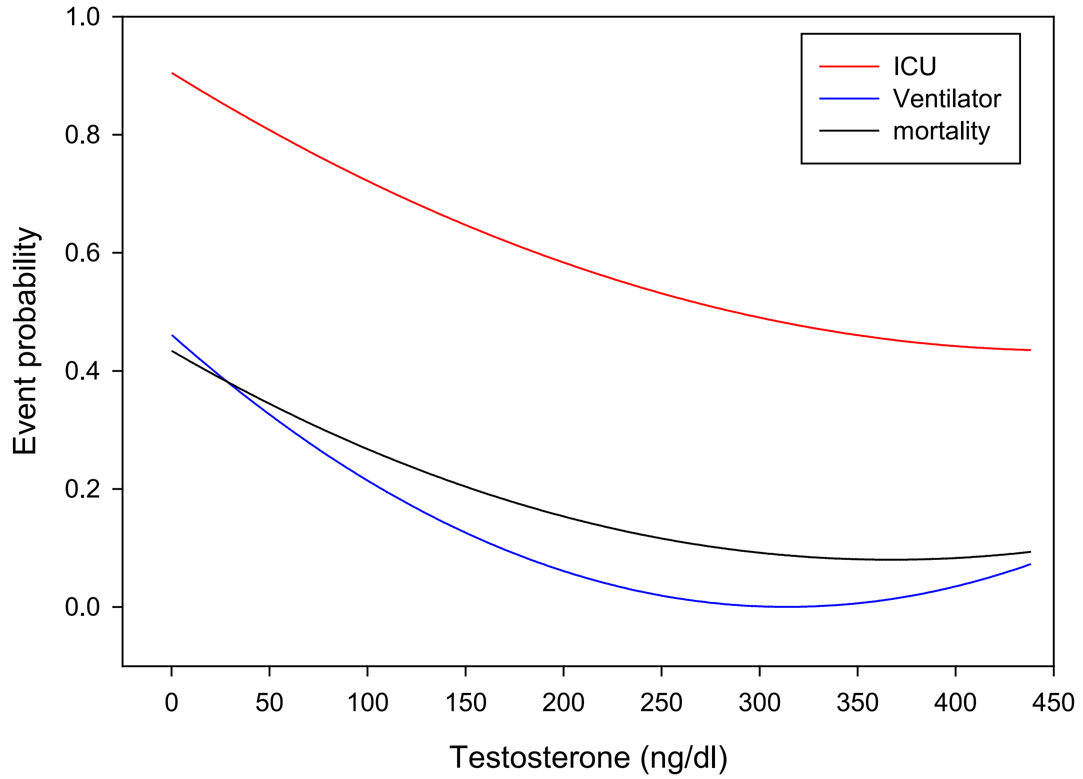

eFigure 5. Regression Curves Demonstrating Probability of Intensive Care Unit Admission, Ventilator Usage, or Mortality as Predicted by Testosterone

After multivariate adjustment for age, BMI, CCI, smoking and race.

© 2021 Dhindsa S et al. *JAMA Network Open.*

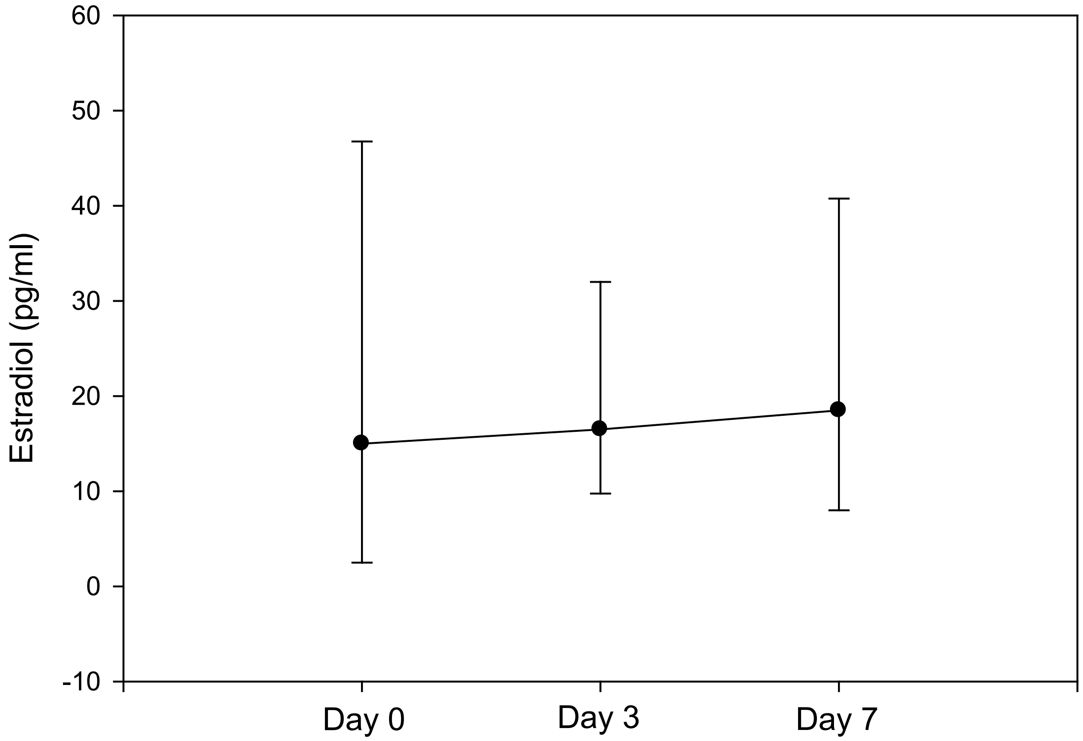

eFigure 6. Estradiol Concentrations in Women at Days 0, 3, and 7

Values are median [25th, 75th percentile]. Estradiol concentrations were available on days 0, 3 and 7 in 44, 26 and 18 patients, respectively.

© 2021 Dhindsa S et al. *JAMA Network Open.*

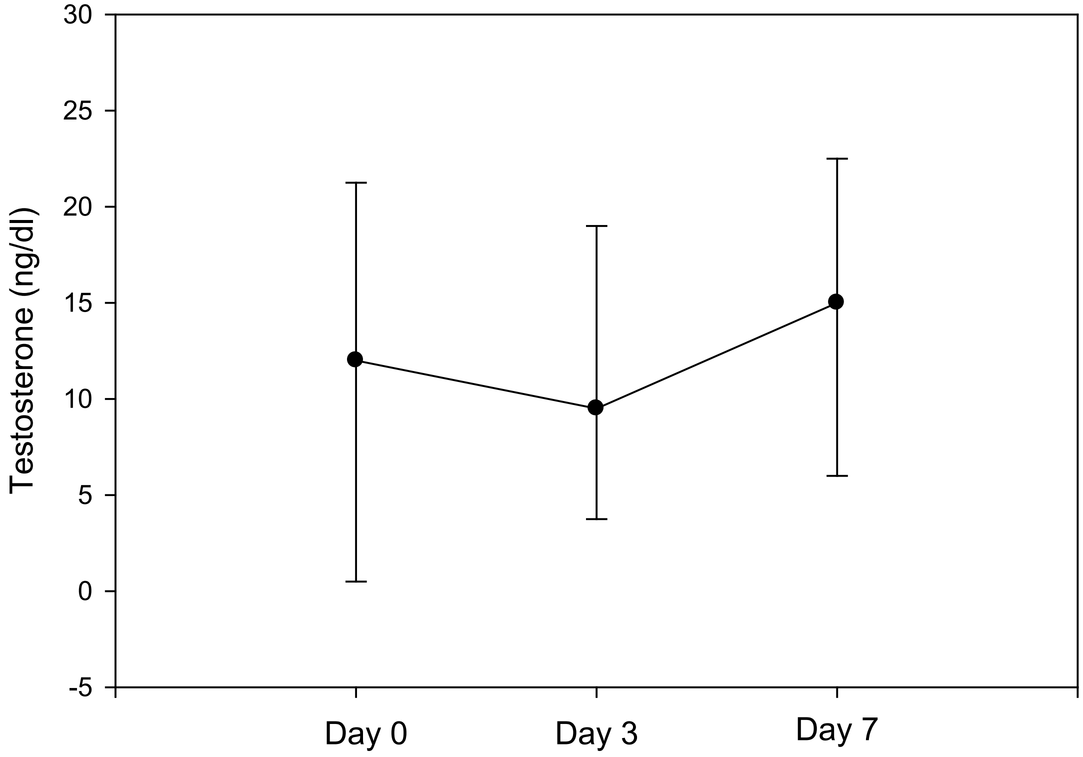

eFigure 7. Testosterone Concentrations in Women at Days 0, 3, and 7

Values are median [25th, 75th percentile]. Testosterone concentrations were available on days 0, 3 and 7 in 56, 30 and 21 patients, respectively.

© 2021 Dhindsa S et al. *JAMA Network Open.*

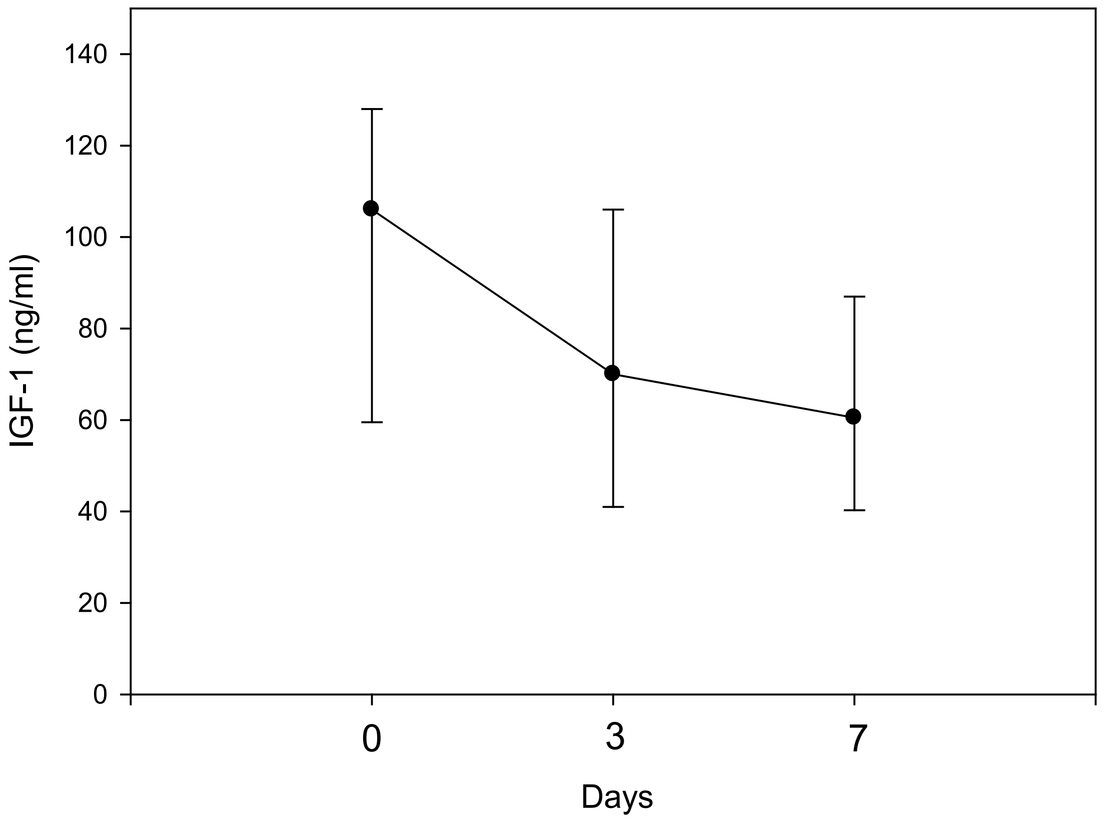

eFigure 8. Insulinlike Growth Factor 1 Concentrations in Women at Days 0, 3, and 7

Values are median [25th, 75th percentile]. IGF-1 concentrations were available on days 0, 3 and 7 in 49, 23 and 14 patients, respectively.

© 2021 Dhindsa S et al. *JAMA Network Open.*

eReferences

1. Colletti JD, Redor-Goldman MM, Pomperada AE, et al. Sample Multiplexing: Increased Throughput for Quantification of Total Testosterone in Serum by Liquid Chromatography-Tandem Mass Spectrometry. *Clinical chemistry*. Sep 1 2020;66(9):1181-1189. doi:10.1093/clinchem/hvaa117
2. Travison TG, Vesper HW, Orwoll E, et al. Harmonized Reference Ranges for Circulating Testosterone Levels in Men of Four Cohort Studies in the United States and Europe. *J Clin Endocrinol Metab*. Apr 1 2017;102(4):1161-1173. doi:10.1210/jc.2016-2935
3. Goldman MM, Clarke NJ, Reitz RE. Methods for Detecting Estradiol by mass Spectrometry. *Patent: US20150309055*.
4. Bystrom CE, Sheng S, Clarke NJ. Narrow mass extraction of time-of-flight data for quantitative analysis of proteins: determination of insulin-like growth factor-1. *Analytical chemistry*. Dec 1 2011;83(23):9005-10. doi:10.1021/ac201800g
5. Bystrom C, Sheng S, Zhang K, Caulfield M, Clarke NJ, Reitz R. Clinical utility of insulin-like growth factor 1 and 2; determination by high resolution mass spectrometry. *PloS one*. 2012;7(9):e43457. doi:10.1371/journal.pone.0043457
6. Dobin A, Davis CA, Schlesinger F, et al. STAR: ultrafast universal RNA-seq aligner. *Bioinformatics (Oxford, England)*. Jan 1 2013;29(1):15-21. doi:10.1093/bioinformatics/bts635
7. Liao Y, Smyth GK, Shi W. featureCounts: an efficient general purpose program for assigning sequence reads to genomic features. *Bioinformatics (Oxford, England)*. Apr 1 2014;30(7):923-30. doi:10.1093/bioinformatics/btt656
8. Love MI, Huber W, Anders S. Moderated estimation of fold change and dispersion for RNA-seq data with DESeq2. *Genome biology*. 2014;15(12):550. doi:10.1186/s13059-014-0550-8
9. Subramanian A, Tamayo P, Mootha VK, et al. Gene set enrichment analysis: a knowledge-based approach for interpreting genome-wide expression profiles. *Proceedings of the National Academy of Sciences of the United States of America*. Oct 25 2005;102(43):15545-50. doi:10.1073/pnas.0506580102
